# Hemorrhage-activated NRF2 in tumor-associated macrophages drives cancer growth, invasion, and immunotherapy resistance

**DOI:** 10.1172/JCI174528

**Published:** 2024-02-01

**Authors:** Dominik J. Schaer, Nadja Schulthess-Lutz, Livio Baselgia, Kerstin Hansen, Raphael M. Buzzi, Rok Humar, Elena Dürst, Florence Vallelian

**Affiliations:** Department of Internal Medicine, University Hospital and University of Zurich, Zurich, Switzerland.

**Keywords:** Inflammation, Oncology, Cancer, Innate immunity, Macrophages

## Abstract

Microscopic hemorrhage is a common aspect of cancers, yet its potential role as an independent factor influencing both cancer progression and therapeutic response is largely ignored. Recognizing the essential function of macrophages in red blood cell disposal, we explored a pathway that connects intratumoral hemorrhage with the formation of cancer-promoting tumor-associated macrophages (TAMs). Using spatial transcriptomics, we found that NRF2-activated myeloid cells possessing characteristics of procancerous TAMs tend to cluster in perinecrotic hemorrhagic tumor regions. These cells resembled antiinflammatory erythrophagocytic macrophages. We identified heme, a red blood cell metabolite, as a pivotal microenvironmental factor steering macrophages toward protumorigenic activities. Single-cell RNA-Seq and functional assays of TAMs in 3D cell culture spheroids revealed how elevated intracellular heme signals via the transcription factor NRF2 to induce cancer-promoting TAMs. These TAMs stabilized epithelial-mesenchymal transition, enhancing cancer invasiveness and metastatic potential. Additionally, NRF2-activated macrophages exhibited resistance to reprogramming by IFN-γ and anti-CD40 antibodies, reducing their tumoricidal capacity. Furthermore, MC38 colon adenocarcinoma–bearing mice with NRF2 constitutively activated in leukocytes were resistant to anti-CD40 immunotherapy. Overall, our findings emphasize hemorrhage-activated NRF2 in TAMs as a driver of cancer progression, suggesting that targeting this pathway could offer new strategies to enhance cancer immunity and overcome therapy resistance.

## Abstract 2

pMicroscopic hemorrhage is associated with the emergence of procancerous tumor-associated macrophages (TAMs), which drive cancer growth, invasion, metastasis, and immunotherapy resistance via heme/NRF2 signaling./p

## Graphical abstract

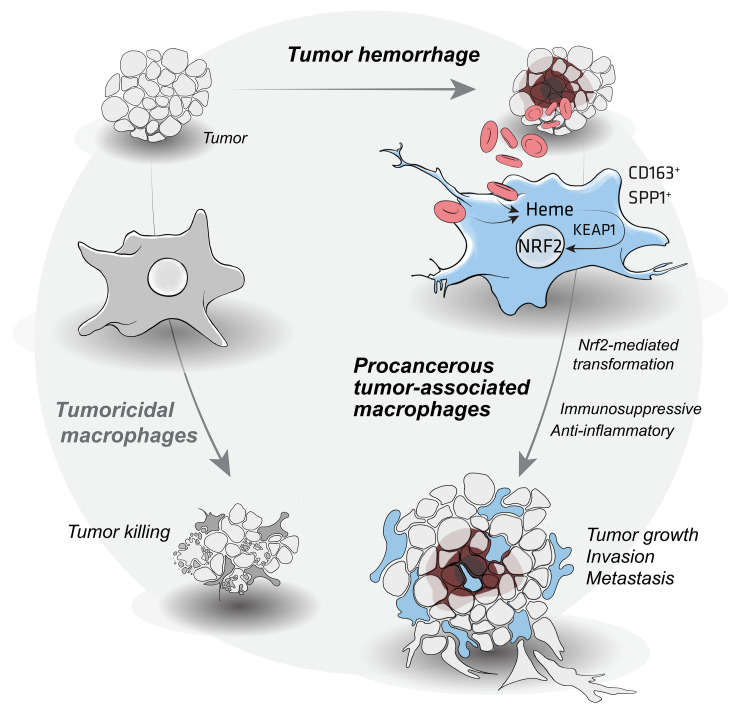

## Introduction

Hemorrhage is omnipresent in solid cancers, occurring at macroscopic and microscopic levels due to invasive tumor growth, pathological vascularization, and therapy-induced or spontaneous necrosis. While traditionally perceived as a disease complication with occasional catastrophic clinical outcomes, the role of hemorrhage as a biological modifier influencing cancer progression and therapeutic responses remains unexplored, creating a unique research gap.

Macrophages, the primary immune cells patrolling the microenvironment of solid cancers, provide an intriguing link between tumor hemorrhage and cancer biology. With their dynamic phenotype and functional versatility, macrophages can respond to diverse spatiotemporal signals to either aid in disease control or contribute to cancer progression (1–4). Tumor-associated macrophages (TAMs) can adopt antitumoral functions when adequately activated by tumor-specific T cells through interferon-γ (IFN-γ) or CD40 signaling (5, 6). Conversely, antiinflammatory cytokines and metabolic stressors, such as fluctuations in pH, electrolyte, or oxygen concentrations, can promote the emergence of procancerous TAMs (7–14). These procancerous TAMs have impaired tumoricidal functions, promote angiogenesis, enable epithelial-mesenchymal transition (EMT), and suppress T cell functions (15–17). The discovery of cellular markers for procancerous TAMs such as SPP1 and CD163 (18–21) in humans and Arg1 (22) in mice has led to extensive research across many cancer types, confirming their association with poor disease outcomes (19, 21–23). As a result, repolarizing of procancerous TAMs into cancer-fighting macrophages, for example, by agonistic anti-CD40 antibodies, presents a promising therapeutic avenue (24, 25).

In healthy and cancerous tissues, macrophages are pivotal in resolving hematomas, ensuring homeostasis, and fostering a microenvironment that promotes tissue repair (26). Macrophages possess unique capabilities to engulf damaged red blood cells (RBCs) and detoxify heme and iron by upregulating heme oxygenase 1 (HMOX1) and antioxidant metabolic pathways regulated by the transcription factor NFE2L2 (NRF2) (27). This macrophage response is considered a “cold” inflammation, because it does not activate an immune response against self-antigens released en masse by damaged cells. However, this response may prove counterproductive in a hemorrhagic tumor by fueling cancer growth in an immunologically cold tumor microenvironment (27, 28).

Our previous work revealed that heme, a metabolite derived from RBCs, activates NRF2 in macrophages, inducing a profound metabolic switch. The NRF2-polarized macrophages undergo reprogramming into erythrophagocytes with a reparative phenotype, compromised immune functions, and remarkable resistance to proinflammatory stimuli (27, 29). Interestingly, these NRF2-polarized erythrophagocytes share phenotypic similarities with procancerous TAMs, such as high HMOX1 and scavenger receptor MARCO expression, while MHC class II is profoundly suppressed. This observation suggests that hemorrhage-derived heme could be an overlooked factor in the cancer microenvironment, promoting the emergence of procancerous TAMs.

Here, we define a trajectory connecting intratumoral hemorrhage to the formation of cancer-promoting TAMs. By analyzing the transcriptomes of human cancers, perinecrotic cancer microenvironments, and artificial hematomas, we discovered substantial similarities between heme-polarized reparative macrophages and cancer-promoting TAMs. Using innovative models to assess TAM functions in 3D cell culture and mice, we demonstrated that upstream heme signaling through NRF2 reprograms macrophages to fuel cancer growth, invasion, metastasis, and resistance to immunotherapy. Targeting and disrupting heme-NRF2 signaling in macrophages may enhance antitumor immune responses and amplify the effectiveness of existing immunotherapies.

## Results

### Tumor hemorrhage connects with procancerous TAM identities.

**Figure 1:**
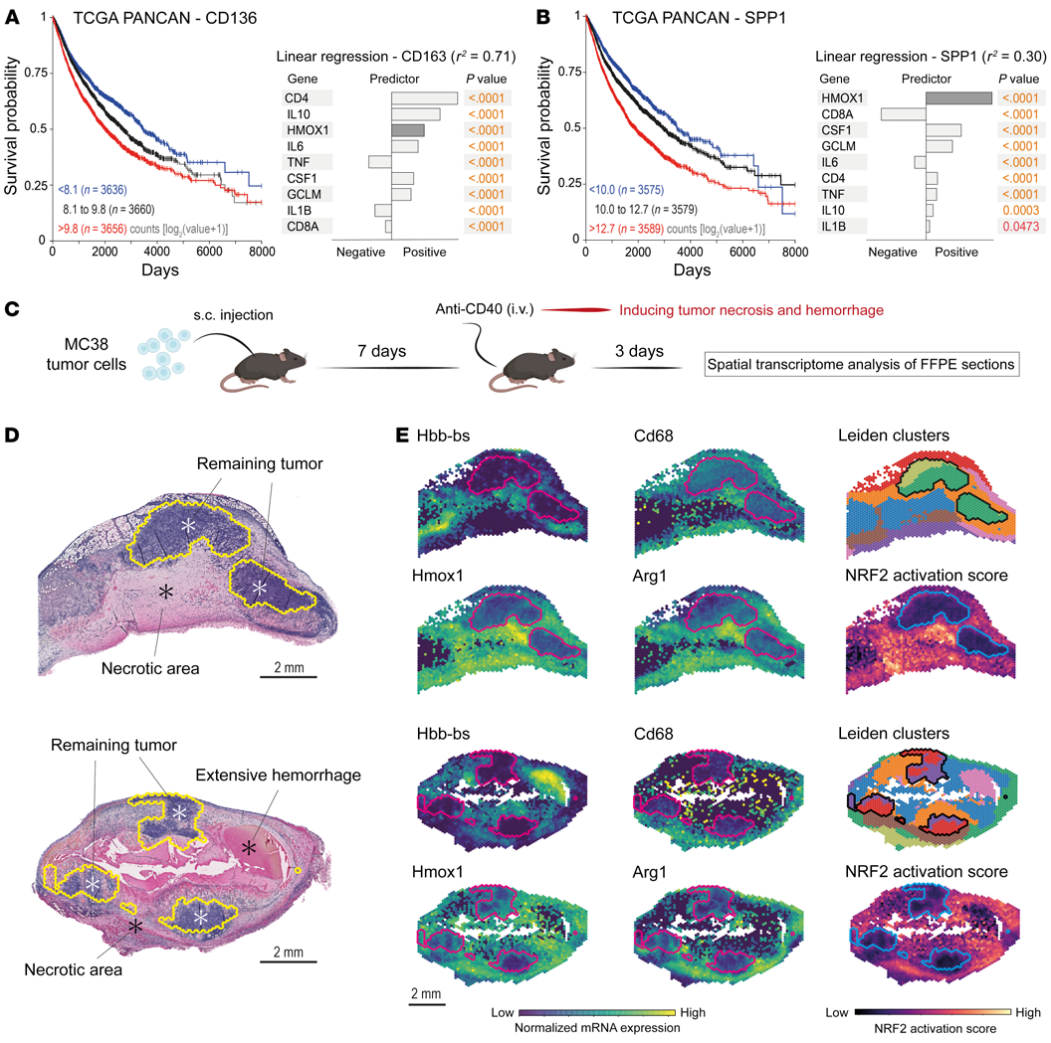
RBC and heme exposure defines an identity of tumor-associated macrophages. (**A**) Left: Kaplan-Meier survival analysis stratified by *CD163* mRNA expression in patients with solid cancers in the TCGA PANCAN database. Right: Linear regression model with *CD163* as the response variable and tissue microenvironmental factors as the predictors (*r*^2^ = 0.71). (**B**) Left: Kaplan-Meier survival analysis stratified by *SPP1* mRNA expression in patients with solid cancers in the TCGA PANCAN database. Right: Linear regression model with *SPP1* as the response variable and the tissue microenvironmental factors as predictors (*r*^2^ = 0.30). (**C**) MC38 tumor–bearing mice were treated with agonistic anti-CD40 antibodies to induce hemorrhagic tumor necrosis. Spatial RNA-Seq analysis was performed to characterize macrophages in the perinecrotic tumor microenvironment. (**D**) HE-stained MC38 tumor sections used for transcriptional analysis. Scale bars: 2 mm. Marked areas indicate the remaining tumoral tissue. (**E**) Expression of selected genes, highlighting the presence of *Cd68^+^Hmox1^+^Arg1^+^* macrophages in the hemorrhagic (Hbb-bs^+^) tumor regions. The *Cd68^+^Hmox1^+^Arg1^+^* regions are marked by a high NRF2 activation score. The lines delineate the remaining tumor.

In the hemorrhagic tissue milieu, macrophages engulf RBCs, leading to pronounced induction of HMOX1 expression (30, 31). Building on this understanding, we postulated that HMOX1 expression within a comprehensive tumor gene expression data set could serve as a marker for investigating the influence of microhemorrhage on the development of CD163^+^ and SPP1^+^ TAMs, which are implicated in tumor progression. First, we analyzed the survival outcomes of over 11,000 patients from The Cancer Genome Atlas (TCGA) Pan-Cancer (PANCAN) database (https://www.cancer.gov/tcga), categorizing them based on their *CD163* and *SPP1* expression levels. Our analysis corroborated the association of elevated *CD163* and *SPP1* levels with diminished survival in patients with solid cancer types. Subsequently, our goal was to construct regression models to evaluate how *HMOX1* expression predicts *CD163* and *SPP1* expression compared with other established TAM modifiers. We compiled a comprehensive list of known macrophage-polarizing stimuli and their representative marker genes from existing literature. This list included T cell markers (*CD4*, *CD8A*), the macrophage growth factor *CSF1*, a spectrum of pro- and antiinflammatory cytokines (*TNF*, *IL1B*, *IL6*, *IL10*), and the oxidative stress marker glutamate-cysteine ligase modifier subunit (*GCLM*). Before modeling, we ensured that the RNA count data for these genes, log_2_-transformed and batch-corrected (log_2_[count + 1]), conformed to a normal distribution. Linear regression models were then developed using *CD163* and *SPP1* as dependent variables, with the microenvironmental factors, including *HMOX1*, as independent variables or predictors (Figure 1, A and B). The model for *CD163* expression demonstrated high accuracy (*r*^2^ = 0.71), with *IL10* and *IL6* positively influencing and *IL1B* and *TNF* negatively influencing *CD163* levels, aligning with existing literature on cytokine impacts on *CD163* expression (32). The model for *SPP1*, though less robust (*r*^2^ = 0.30), was still statistically significant. The negative correlation of *SPP1* expression with *CD8A* further supports the role of SPP1^+^ TAMs in immune suppression (33). *HMOX1* was identified as a strong positive predictor for both TAM markers, suggesting that intratumoral heme exposure due to microhemorrhage may be a critical factor in fostering cancer-promoting TAMs.

Next, we aimed to identify TAM phenotypes in hemorrhagic tumors. We established an experimental model of therapy-induced hemorrhagic tumor necrosis by treating GFP-MC38 tumor–bearing mice with a single injection of the anti-CD40 agonist antibody FGK4.5 seven days after subcutaneous tumor cell injection (Figure 1C). We selected the MC38 colon adenocarcinoma model because these tumors are known to be sensitive to anti-CD40 immunotherapy (34). Three days after the anti-CD40 antibody injection, the tumors were collected for histology and genome-wide spatial analysis of mRNA expression. In tumor samples from 2 mice, HE histology revealed extensive tumor necrosis with RBC extravasation into the necrotic areas (Figure 1D), thus confirming the presence of hemorrhagic tumor tissue. Then, we performed spatial gene expression analysis to achieve more detailed phenotyping; this analysis discriminated zones of remaining viable cancer cells surrounded by hemorrhagic necrosis, as evidenced by the enrichment of RBC-derived mRNA (*Hbb-bs*) (Figure 1E). In these hemorrhagic regions, we found an increased *Cd68* signal, confirming macrophage infiltration, superimposed by upregulated expression of *Hmox1* and *Arg1*, the latter of which is the archetypal marker for procancerous and immunosuppressive TAMs in mice (22), similar to *CD163* and *SPP1* in human cancers.

Finally, since key characteristics of antiinflammatory erythrophagocytic macrophages are driven by activated NRF2, we plotted an expression score of NRF2-activated genes. The results suggested high NRF2 activity in the *Cd68*^hi^*Hmox1*^hi^*Arg1*^hi^ tumor regions (Figure 1E). In summary, these data indicate that hemorrhage enforces the accumulation of macrophages with a phenotype consistent with immunosuppressive and procancerous TAMs.

### RBCs and heme drive the generation of TAM-like macrophages with an immunocompromised phenotype.

**Figure 2:**
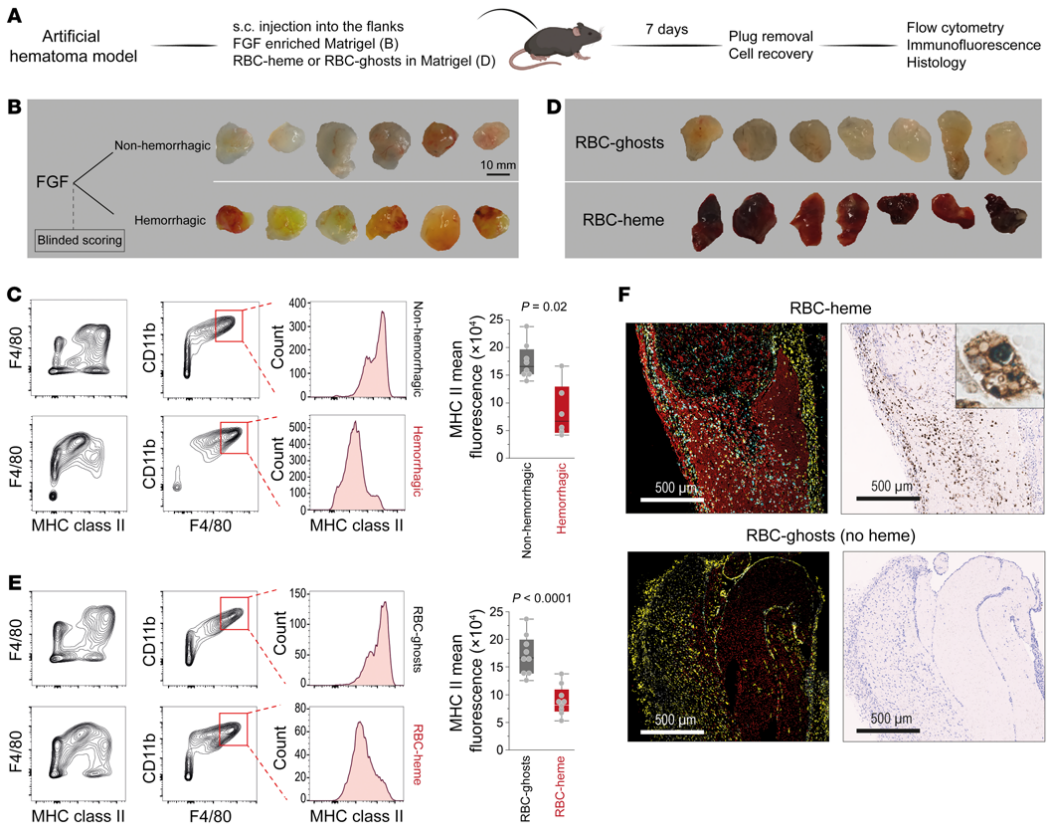
Erythrophagocytic transformation of macrophages in RBC-heme Matrigel plugs. (**A**) Subcutaneously placed FGF-enriched Matrigel plugs were used to study the effect of hemorrhage on the phenotype of invading macrophages. Plugs enriched with intact RBCs (RBC-heme) or RBC-ghosts were used to study the specific effect of hemoglobin-heme. (**B**) FGF-enriched Matrigel plugs were collected from the subcutaneous (s.c.) injection site 7 days after injection and classified by blinded investigators as non-hemorrhagic or hemorrhagic based on their macroscopic appearance. (**C**) Flow cytometry contour plots of plug-invading cells, defining F4/80^+^CD11b^+^ macrophages with lower MHC class II expression in hemorrhagic plugs. The box plots depict the mean fluorescence intensities of MHC class II expression within the CD11b^+^F4/80^+^ population (*n* = 6–8 plugs per condition; each dot represents 1 plug; *t* test). (**D**) RBC-heme and RBC-ghost plugs were collected 7 days after s.c. injection. (**E**) Flow cytometry contour plots of plug-invading cells defining F4/80^+^CD11b^+^ macrophages with lower MHC class II expression in RBC-heme than RBC-ghost plugs. The box plots depict the mean fluorescence intensities of MHC class II expression within the CD11b^+^F4/80^+^ population (*n* = 9 plugs per condition; each dot represents 1 plug; *t* test). (**F**) Fluorescence immunohistochemistry images of Matrigel plug sections stained with TER119 (red, RBC-heme or RBC-ghost), anti-F4/80 (yellow, macrophages), and anti-HMOX1 antibodies (cyan). Nuclei were stained with DAPI (white). Images were acquired using a PhenoImager HT (Akoya). Corresponding consecutive sections stained for iron show iron accumulation in infiltrating cells. Scale bars: 500 μm. The inset shows an iron-positive erythrophagocyte containing multiple RBC remnants.

Experimentally manipulating the tumor microenvironment to study phenotype-driving factors poses substantial challenges. Hence, we examined macrophages within modifiable artificial tissue scaffolds based on Matrigel plug constructs, which we subcutaneously placed in mice (Figure 2A). In the first model, we focused on the effect of a hemorrhage on macrophage phenotypes. We enriched Matrigel with fibroblast growth factor (FGF), resulting in variable plug vascularization and hemorrhage. Seven days after subcutaneous injection, plugs were explanted, and blinded investigators classified them as non-hemorrhagic (white/gray appearance) or hemorrhagic (red/yellow appearance, indicating the presence of hemoglobin, heme, and bilirubin) (Figure 2B). For both conditions, flow cytometry revealed infiltration of CD11b^+^F4/80^+^ macrophages. However, the degree of MHC class II expression was significantly lower in the hemorrhagic compared with the non-hemorrhagic plugs (Figure 2C). This observation suggests that macrophages accumulating in a hemorrhagic tissue microenvironment exhibit an immunocompromised phenotype.

In the second set of experiments, we focused on the specific role of RBC-derived heme as an inducer of this MHC class II^lo^ macrophage phenotype by incorporating mouse RBCs (RBC-heme) or heme-depleted RBC-ghosts into the Matrigel (Figure 2D). Again, we detected CD11b^+^F4/80^+^ macrophages in both plug types. MHC class II expression was lower in the RBC-heme plugs (Figure 2E). In addition, multiplexed-fluorescence immunohistochemistry demonstrated that the infiltrating macrophages in the RBC-heme plugs were HMOX1 positive and iron loaded, consistent with a post-erythrophagocytic state, which was observed at higher magnification by the visualization of ingested RBC remnants (Figure 2F and Supplemental Figure 1A; supplemental material available online with this article; https://doi.org/10.1172/JCI174528DS1). This suggests that erythrophagocytosis is the principal route of heme delivery into the cells. Compared with the heme concentration within RBCs (~18 mM), the cell-free hemoglobin and heme concentrations in the plugs were low (Supplemental Figure 1, B and C).

**Figure 3:**
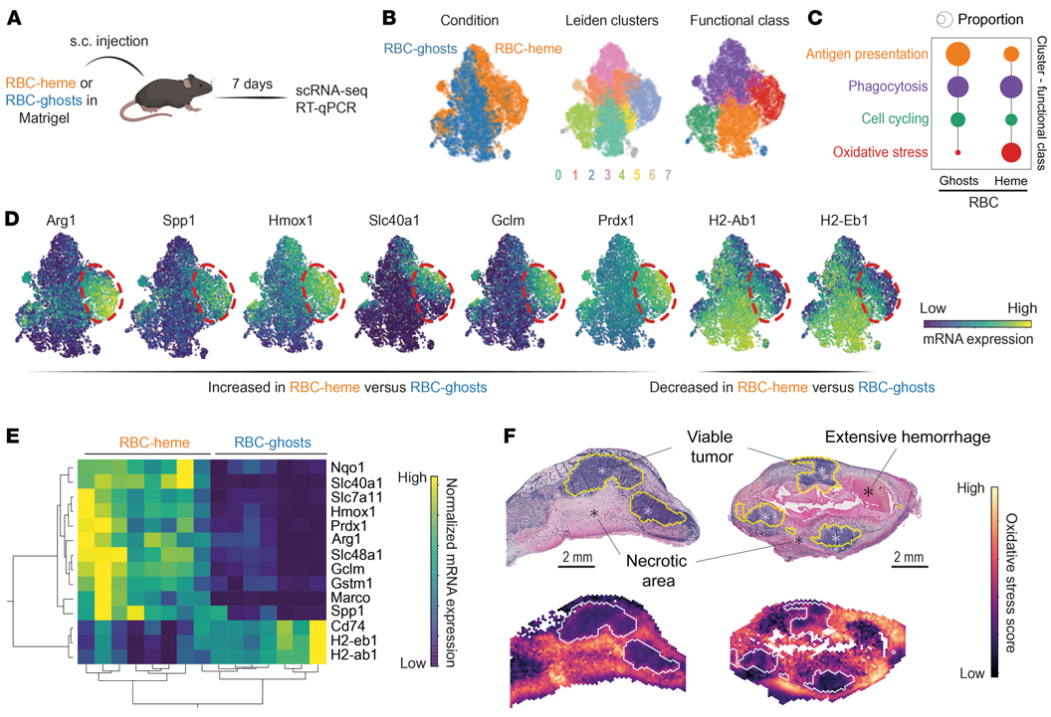
RBC-heme enforces the transformation of macrophages into heme-TAMs. (**A**) RBC-heme– and RBC-ghost–enriched Matrigel plugs were collected 7 days after s.c. placement and processed for scRNA-Seq analysis. (**B**) Plug-invading cells were enriched by anti-F4/80 and anti-CD11b magnetic beads from an RBC-heme and an RBC-ghost Matrigel plug before scRNA-Seq analysis. UMAPs are color-coded for condition, Leiden cluster, and the attributed functional class. (**C**) Proportion of macrophages per functional class stratified for RBC-heme and RBC-ghost. The dot size corresponds to the proportion of cells (%) per functional class. This analysis visualizes that erythrophagocytosis fosters the emergence of oxidative stress–handling macrophages while suppressing cells equipped for antigen presentation. (**D**) Expression heatmaps of selected signature genes. The dashed line highlights the region of the UMAP containing the oxidative stress cluster. These macrophages have high expression of *Arg1*, *Spp1*, and heme-, iron-, and oxidative stress–handling genes, while expression of MHC class II–related genes is low. (**E**) Expression heatmap and unsupervised hierarchical clustering analysis of heme-TAM marker genes measured by quantitative reverse transcriptase PCR (RT-qPCR) in plug-invading cells after enrichment of F4/80^+^ cells. Each column represents data from 1 plug collected 7 days after s.c. injection (*n* = 8 RBC-heme plugs, *n* = 7 RBC-ghost plugs). (**F**) A score for the oxidative stress–related macrophages in the RBC plug was calculated based on differentially expressed genes (log_2_[fold change] 2, *P* 0.001, *n* = 114 genes). This score was mapped into the spatial transcriptome data of the MC38 tumor sections reported in Figure 1. The heatmap visualizes that macrophages with an oxidative stress–handling identity accumulate in the perinecrotic tumor microenvironment.

To further delineate whether these RBC-heme–transformed macrophages share the phenotypic features of procancerous TAMs, we recovered macrophages from collagenase-digested RBC-heme or RBC-ghost plugs with F4/80 antibody–coupled magnetic beads and performed single-cell RNA-Seq (scRNA-Seq) analysis (Figure 3A). We identified 6,434 macrophages (Figure 3B) and functionally classified them using published gene sets for specific macrophage functions (35). This analysis assigned the 7 Leiden clusters to 4 major functional groups: phagocytosis, oxidative stress, antigen presentation, and cell cycling (Figure 3B and Supplemental Figure 1D). The category distributions are consistent with the pathway score intensities per cell. The predominant effect of heme-containing RBCs compared with RBC-ghosts was the emergence of a large population of macrophages attributed to the oxidative stress state with high expression of NRF2 target genes and a reduction in cells designated for antigen presentation (Figure 3C and Supplemental Figure 1E).

Heme-exposed macrophages in RBC-heme plugs were characterized by high expression of genes related to the metabolism of heme (*Hmox1*), iron (*Slc40a1*), and antioxidants (*Gclm*, *Prdx1*). They also expressed the procancerous TAM markers *Arg1* and *Spp1* (Figure 3D). In contrast, MHC class II expression (*H2-Ab1*, *H2-Eb1*) was diminished within these macrophages. The different macrophage phenotypes in RBC-heme plugs versus RBC-ghost plugs were confirmed in independent experiments by quantitative reverse transcriptase PCR analysis of selected signature genes (Figure 3E). These data support that heme exposure leads to the emergence of *Arg1*^hi^*Spp1*^hi^ macrophages.

Finally, we projected the gene expression score defining the RBC-heme–induced *Arg1*^hi^*Spp1*^hi^ oxidative stress macrophages into the spatial gene expression map of the hemorrhagic GFP-MC38 tumors. We found a high score intensity in the hemorrhagic necrosis zones around the viable cancer tissue remnants (Figure 3F). This supports the idea that the macrophages in our Matrigel scaffold model represent hemorrhage-associated TAMs.

Collectively, these data suggest that the sequence of RBC extravasation, erythrophagocytosis, and intracellular heme signaling generates *Arg1*^hi^*Spp1*^hi^ macrophages. These cells exhibit an NRF2-regulated antioxidant gene expression profile and attenuated immune function, sharing strong phenotypic characteristics with procancerous TAMs. From this point forward, we refer to these macrophages as heme-TAMs.

### Mixed-cell-type spheroids support the generation and maintenance of heme-TAMs in an experimental tumor microenvironment.

**Figure 4:**
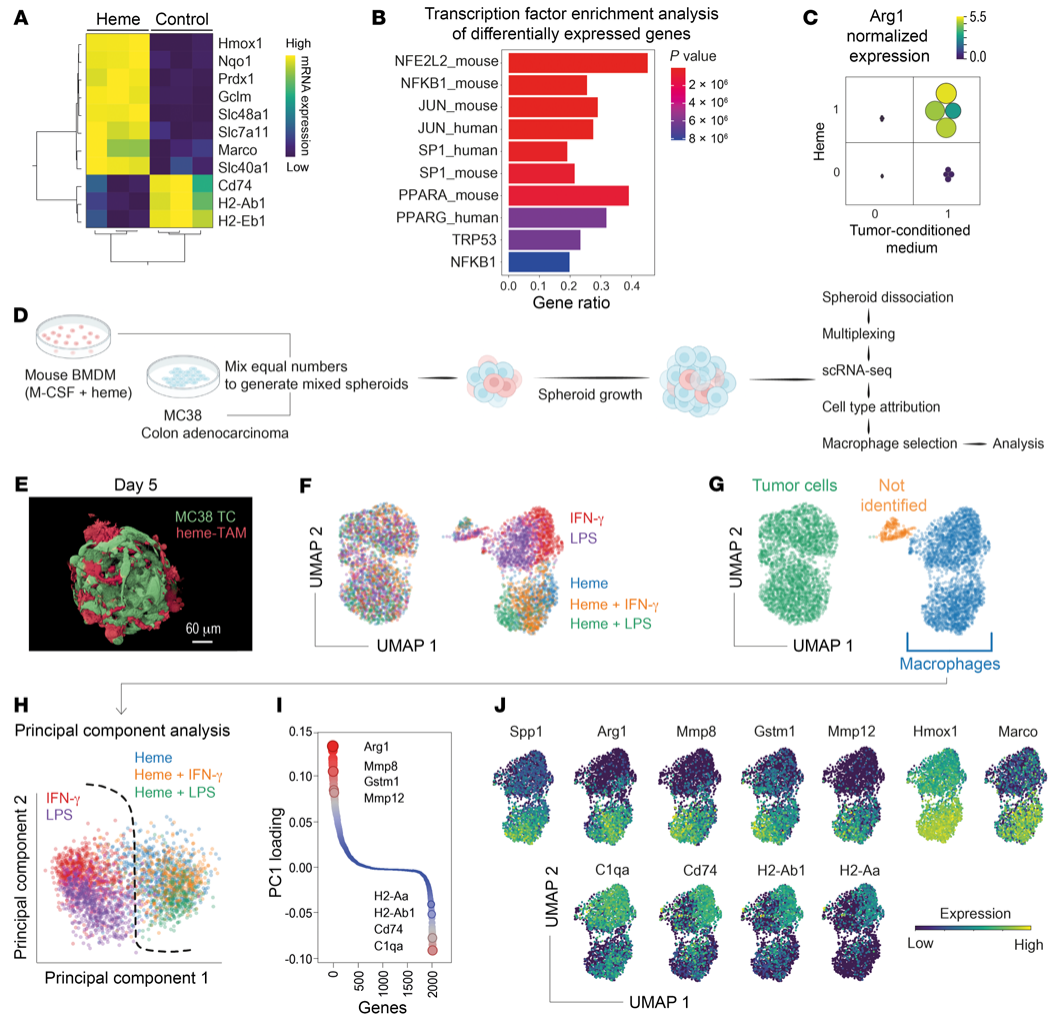
Heme-TAMs in 3D spheroid cancer cell cultures. (**A**) Expression heatmap and unsupervised hierarchical clustering analysis of marker genes quantified by bulk RNA-Seq in control and heme-treated BMDMs cultured in 2D (normalized log_2_ counts). Each column represents macrophages from 1 mouse (*n* = 3). (**B**) EnrichR analysis of all significantly differentially expressed genes (log_2_[fold change] 0.5, *P* 0.001, *n* = 3). The gene ratio defines the overlap of the input genes and the term-associated genes, and the top 10 enriched terms are shown. Terms are ranked by their *P* value. (**C**) Results of a factorial experiment defining the synergistic effect of heme and MC38 cell culture supernatant on *Arg1* mRNA expression in 2D BMDM cultures. *Arg1* expression was measured by RT-qPCR. Color and size of the dots represent the normalized gene expression per sample (*n* = 4 BMDM cultures per condition). (**D**) Experimental workflow used to generate and analyze mixed-cell-type 3D spheroids containing MC38 tumor cells and BMDMs. (**E**) 3D reconstruction of a confocal microscopy image stack of a mixed-cell-type spheroid resulting from a 5-day culture of GFP-MC38 cells (green) and heme-TAM-tomato cells (red) in a microwell plate. Scale bar: 60 μm. (**F**) Results of a multiplexed scRNA-Seq experiment with mixed-cell-type spheroids containing MC38 cells and BMDMs that had been pretreated with heme, LPS, IFN-γ, or combinations thereof. The spheroids were collected for scRNA-Seq 24 hours after the 2 cell types were mixed and seeded on microwell plates. The UMAP is color-coded to indicate the BMDM pretreatment. (**G**) Cell-type assignment of the spheroid cells was performed, and macrophages were extracted for further analysis. (**H**) In PCA of spheroid macrophages, PC1 segregates the heme-pretreated BMDMs from those not pretreated with heme. (**I**) The contribution of individual genes to PC1 is expressed as loadings. Genes are ordered and color-coded by their PC1 loading. A high absolute value indicates that the gene strongly influences overall gene expression variance. The highest PC1 loading was found for *Arg1*. (**J**) Expression heatmaps of the top PCA drivers and selected heme-induced TAM marker genes.

In the next step, we sought to develop a model to facilitate the generation and functional characterization of heme-TAMs in vitro. To do this, we tested whether treating bone marrow–derived macrophages (BMDMs) with heme in cell culture would transform them into heme-TAMs. Bulk RNA-Seq (Figure 4A) revealed that heme exposure induced a *Hmox1*^hi^*Marco*^hi^*MHC class II*^lo^ macrophage phenotype and strong expression of NRF2-regulated genes (Figure 4B). However, these cells showed weak expression of the TAM marker gene *Arg1*, indicating that heme treatment alone was insufficient to induce the full heme-TAM phenotype. Therefore, we explored whether additional tissue microenvironmental factors could synergize with heme. In a factorial study, we treated BMDMs with heme, MC38 colon adenocarcinoma cell culture supernatant, or a combination of both stimuli. Although neither of the 2 stimuli alone could fully induce the expression of *Arg1*, we found a strong synergistic effect (Figure 4C), suggesting that heme, in conjunction with factors provided by the tumor cell culture supernatant, serves as a potent signal for the generation of heme-TAMs.

Next, we established a 3D culture of MC38 cancer cells mixed with heme-treated BMDMs in microwell plates to mimic the tumor microenvironmental TAM niche more accurately (Figure 4D). This procedure generated tumor spheroids with uniformly interspersed macrophages (Figure 4E). We profiled the transcriptome of macrophages within these spheroids using scRNA-Seq 24 hours after spheroid formation. To test the robustness of heme as a TAM phenotype driver, we conducted a multiplexed experiment evaluating different macrophage pretreatments consisting of heme alone and combinations of heme with the classical M1-polarization stimuli IFN-γ and LPS (Figure 4F). Macrophages were identified by *Ptprc* (CD45) expression (Figure 4G) and subjected to principal component analysis (PCA) based on the highly variable genes (Figure 4H and Supplemental Figure 2A). PC1 separated all heme-exposed macrophages from cells treated solely with IFN-γ or LPS, describing a dominant heme effect that superseded the impact of the proinflammatory stimuli. Analysis of all mRNA transcripts sorted by their weighted contribution to PC1 (PC1 loading) revealed that high expression of the TAM marker gene *Arg1*, NRF2 target genes (e.g., *Gstm1*), and TAM-associated matrix remodeling genes (*Mmp8*, *Mmp12*) was highly discriminative, as was suppression of MHC class II genes (*H2-Ab1*, *H2-Aa*), *Cd74*, and *C1qa* (Figure 4I). Moreover, macrophages exposed to heme had higher expression levels of *Hmox1*, *Marco*, and the TAM marker *Spp1* (Figure 4J). Before conducting functional studies, we wanted to confirm the stability of the heme-TAM phenotype during extended spheroid culture. Therefore, we analyzed macrophages extracted from spheroids after 4, 8, and 10 days (Supplemental Figure 2, B and C). Based on expression data for *Marco*, *Arg1*, *Spp1*, *Cd74*, and *H2-Ab1*, the macrophage phenotype was remarkably stable (Supplemental Figure 2D).

In summary, we discovered that within the microenvironment of a spheroid culture system, heme exposure drives the transformation of macrophages into *Arg1*^hi^*Spp1*^hi^ heme-TAMs with an enhanced matrix remodeling program and suppressed immune marker genes. Furthermore, heme-TAMs resist inflammatory rewiring by IFN-γ and LPS.

### Heme-TAMs prevent cancer cell apoptosis and drive growth, invasiveness, and metastasis.

**Figure 5:**
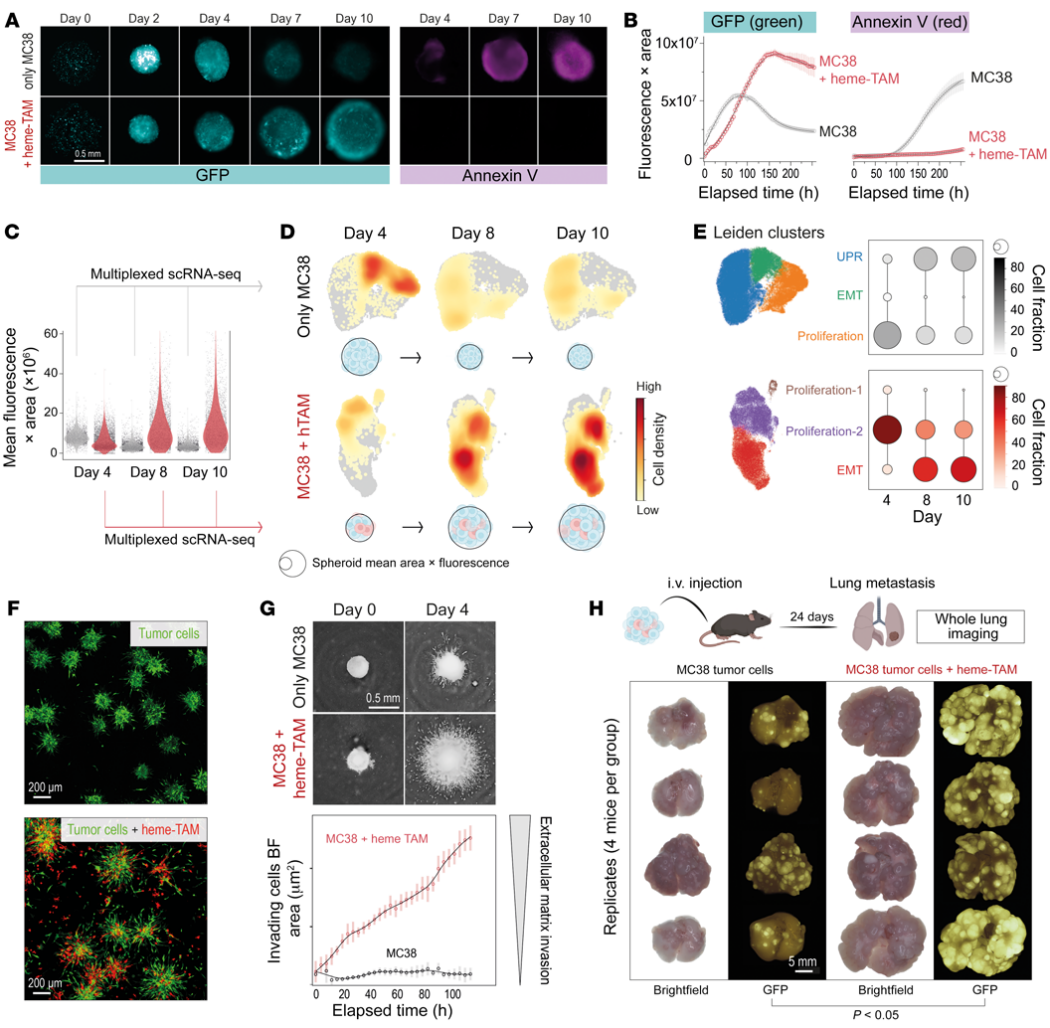
Heme-TAMs support tumor cell growth, invasiveness, and metastasis. (**A**) Spheroid GFP fluorescence (magenta) and annexin V (cyan). (**B**) Integrated fluorescence across the spheroid area. Data are mean ± 95% CI of 42 replicates. (**C**) Spheroids were grown in microwell plates for scRNA-Seq experiments and GFP fluorescence across the spheroid area was quantified for ≥3,000 spheroids per condition and used for cell density correction in **F** (gray, MC38; red, MC38+heme-TAMs) (ANOVA with Tukey-Kramer post-test corrected with *P* 0.001 for all comparisons, except MC38 day 8 vs. MC38 day 10, *P* = 0.99). (**D**) Multiplexed scRNA-Seq of MC38 tumor cell spheroids (only MC38) or mixed-cell-type spheroids (MC38 + heme-TAM) on days 4, 8, and 10 after formation. After macrophage exclusion, cell densities were scaled by the mean spheroid size before projecting on the UMAP. (**E**) Leiden clustering defined 3 clusters per experiment, a dominant functional annotation for each cluster was determined by GSEA. Dot plots depict the fraction of tumor cells within each functional state per time point. (**F**) GFP-MC38 spheroids (top) and mixed GFP-MC38+heme-TAM-tomato spheroids (bottom) were transferred from microwell plates to a flat glass-bottom plate on day 4 after spheroid formation and embedded into an extracellular matrix. Cell invasion was imaged 24 hours later. (**G**) Four days after formation, spheroids were embedded into an extracellular matrix (*t* = 0 hours), and cell invasion was measured by live-cell imaging every 4 hours. Top: Representative inverted bright-field images. Scale bar: 0.5 mm. Bottom: The invading cell front was automatically segmented and quantified over time. Data are mean ± 95% CI of 21 replicates analyzed within 1 representative experiment. (**H**) Spheroids were collected on day 5 after formation and injected i.v. into C57BL/6J mice. Lungs were collected 24 days after injection. *t* test. Scale bar: 5 mm.

We next established an experimental framework to study the impact of heme-TAMs on growth and phenotype of cancer cells (Supplemental Figure 3A). In the first step, we cultured GFP-MC38 cells alone or with heme-treated macrophages in 96-well ultra-low-attachment plates. We monitored spheroid growth and apoptosis as GFP fluorescence and annexin V accumulation using a live-cell imaging system. In the tumor-cell-only spheroids, we observed a decay in GFP fluorescence after day 4, coinciding with increasing annexin V fluorescence. In contrast, GFP fluorescence progressively increased without an annexin V signal in the mixed-cell-type spheroids (Figure 5, A and B). This was consistent with ATP measurements in single spheroids, which decreased after day 4 in the tumor-cell-only spheroids but increased in the mixed-cell-type spheroids (Supplemental Figure 3B). In a complementary setup, we cultured the same cells in microwell plates and collected numerous uniform spheroids for metabolic flux analysis at different time points. Again, on days 8 and 10 in culture, tumor-cell-only spheroids decayed metabolically, while the mixed-cell-type spheroids demonstrated an unaltered oxygen consumption rate throughout the 10-day culture period (Supplemental Figure 3C). Collectively, we observed that after 4 days, the size of GFP-MC38 spheroids regressed, and the tumor cells underwent apoptosis and decayed metabolically. In contrast, the presence of heme-TAMs within spheroids prevented apoptosis, supporting the extended growth of metabolically viable spheroids up to 10 days of culture.

To understand the impact of heme-TAMs on the cancer cell phenotype, we conducted multiplexed scRNA-Seq experiments with tumor-only and mixed-cell-type spheroids collected on days 4, 8, and 10 after formation. Before we processed the cells for scRNA-Seq, we measured the size and fluorescence intensity of each analyzed spheroid by fluorescence microscopy to confirm the heme-TAM–supported spheroid growth (Figure 5C). After quality control and demultiplexing, macrophages were excluded from the analysis, and Leiden clustering of the tumor cells defined 3 clusters per condition (Supplemental Figure 3D). Cell density projections illustrate how the densities migrate through the uniform manifold approximation and projection (UMAP) across time, indicating that the transcriptome undergoes substantial changes (Figure 5D). We then used gene set enrichment analysis (GSEA) to extract the dominant functional characteristics for each cluster. The most significantly enriched pathways were related to proliferation, EMT, and the unfolded protein response (UPR) (Supplemental Figure 3D). An analysis of the proportion of tumor cells attributed to each cluster/functional category on days 4, 8, and 10 revealed that tumor cell spheroids were proliferative and in a state of EMT on day 4 but entering the UPR state afterward, with most cells projecting into the UPR cluster on days 8 and 10 (Figure 5E). This is consistent with the progressive apoptosis and metabolic decay of these spheroids. In contrast, tumor cells in the mixed spheroids were predominantly proliferative on day 4, transitioning into a stable state of proliferation and EMT. The expression of selected signature genes for proliferation, EMT, and UPR is highlighted in Supplemental Figure 3E.

EMT is a physiological process that enhances invasiveness and is thus a key determinant of cancer progression (36). In mixed spheroids, heme-TAMs dramatically increased the invasion of GFP-MC38 tumor cells, which progressed for more than 4 days after the spheroids were embedded into an extracellular matrix (Figure 5, F and G). The ultimate disease correlate of EMT is cancer metastasis. Consequently, we collected spheroids 5 days after formation and injected approximately 750 intravenously into mice. Three weeks after injection, we observed substantially more extensive pulmonary metastasis in mice injected with mixed spheroids than in those injected with tumor cell spheroids (Figure 5H). Taken together, our findings suggest that heme-TAMs promote tumor cell proliferation and EMT, fostering an invasive and highly metastatic cancer phenotype.

### Heme-TAMs resist tumoricidal transformation by IFN-γ.

**Figure 6:**
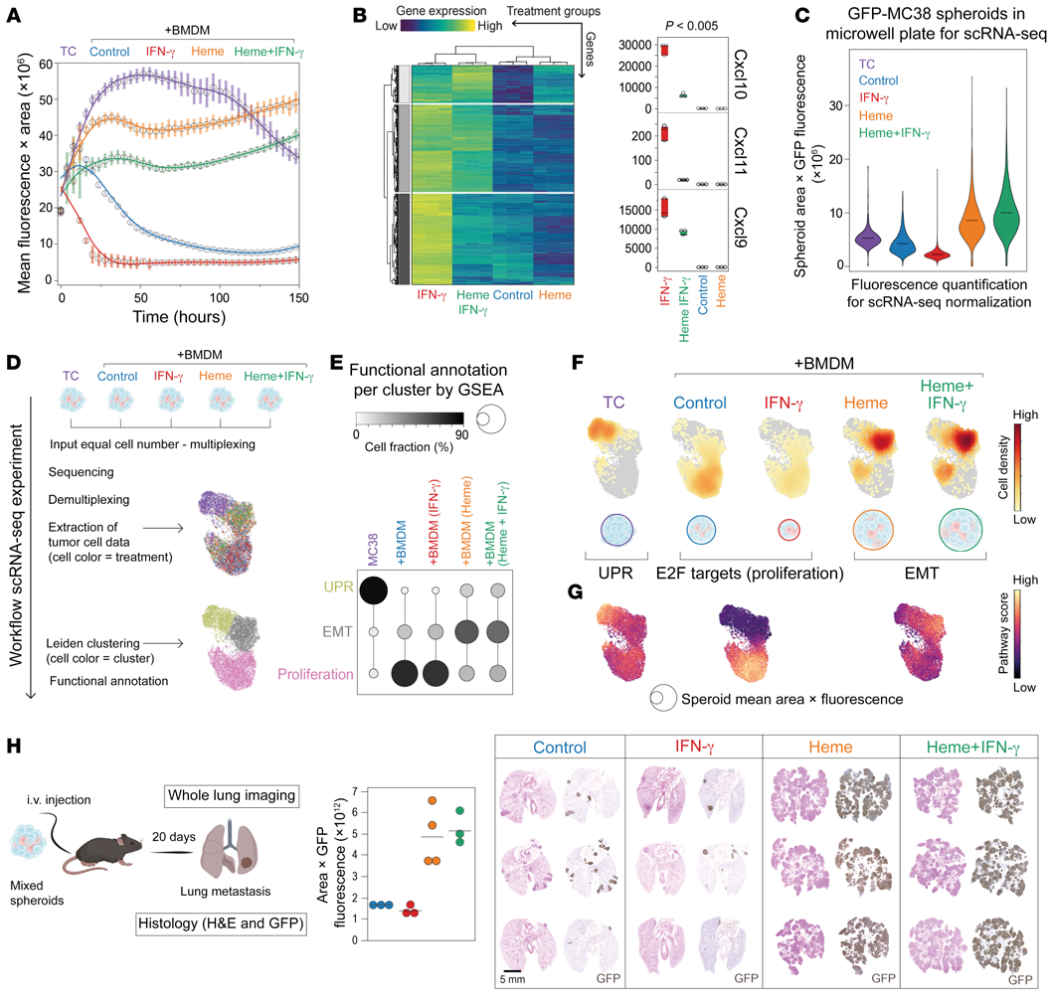
Heme-TAMs resist tumoricidal transformation by IFN-γ. (**A**) Integrated spheroid GFP fluorescence obtained by live-cell microscopy. Data are mean ± 95% CI of 10 replicates from 1 representative experiment. (**B**) Expression heatmap and clustering of differentially expressed genes (log_2_[fold change] 0.5, *P* 0.005, *n* = 3). Right: Normalized count data for *Cxcl9*, *Cxcl10*, and *Cxcl11*. Each point represents 1 replicate. (**C**) Spheroids identical to those in **A** were grown in microwell plates for scRNA-Seq and scanned with a fluorescence microscope on day 9. Violin plots depict GFP fluorescence integrated across the object area for ≥1,400 spheroids per condition (ANOVA with Tukey-Kramer post-test corrected with *P* 0.001 for each comparison). (**D**) scRNA-Seq workflow. (**E**) GSEA-defined functional attributes for the 3 tumor cell clusters. Dot plot visualizes the fraction of tumor cells within each functional state. (**F**) Tumor cell densities normalized by the mean integrated fluorescence of the input. Bubbles beneath the UMAPs depict the mean spheroid size. (**G**) Gene expression score intensities for GSEA categories. (**H**) Approximately 750 spheroids (GFP-MC38 cancer cells + BMDMs) were collected from microwell plates on day 4 after spheroid formation and injected i.v. into *Rag2^−/−^γc^−/−^* mice. Lungs were collected 20 days after injection. Paraffin sections of lung tissue visualize metastatic disease. Scale bar: 5 mm. GFP fluorescence of whole-lung fluorescence images (see Supplemental Figure 4E) was integrated across the imaged lung area and quantified for *n* = 3–4 animals per condition. Each dot represents 1 mouse (lung). ANOVA with Tukey-Kramer post-test corrected for multiple comparisons, IFN-γ vs. heme + IFN-γ *P* = 0.0028, heme + IFN-γ vs. control *P* = 0.0046, heme vs. IFN-γ *P* = 0.0030, heme vs. control *P* = 0.0051, control vs. IFN-γ *P* = 0.98, heme vs. heme + IFN-γ *P* = 0.97.

Inflammatory macrophage activation in the tumor microenvironment is a critical component of the antitumor immune response and an emerging therapeutic concept (25). So far, we have focused our functional studies on the procancerous effects of heme-TAMs, ignoring the comparison with other macrophage polarization states. We mixed GFP-MC38 cells with BMDMs that were either untreated, representing an undetermined polarization state, or pretreated with heme, IFN-γ, or heme plus IFN-γ, and monitored spheroid growth and regression over time (Figure 6A). Our findings showed that untreated macrophages had a tumoricidal effect immediately after spheroid formation, which IFN-γ further enhanced. In contrast, heme-pretreated BMDMs were not tumoricidal and instead promoted tumor cell growth, as seen before. This pattern of spheroid growth was not altered when heme-TAMs were treated with IFN-γ, suggesting that heme-TAMs were resistant to the tumoricidal effect of IFN-γ. This is consistent with a bulk RNA-Seq experiment with BMDMs, which demonstrated that heme substantially repressed the IFN-γ–induced transcriptional response. This repression included *Cxcl9*, *Cxcl10*, and *Cxcl11*, which have been identified as prognostically favorable markers of an antitumor microenvironment (18) (Figure 6B).

We repeated the identical experiment using scRNA-Seq as a readout to obtain more in-depth insight into the effects of differently polarized BMDMs on MC38 tumor cells. We stimulated BMDMs with heme, IFN-γ, or heme plus IFN-γ, and measured mitochondrial function and the glycolytic rate of BMDMs using a Seahorse metabolic flux analyzer (Seahorse XFe24 Analyzer, Agilent) to confirm maintained viability (Supplemental Figure 4A). Spheroids were then generated and cultured in microwell plates. Before spheroids were dissociated and labeled for multiplexing, the size and fluorescence intensity of each spheroid were quantified on day 9. These data confirmed the enhanced tumoricidal activity of IFN-γ–treated BMDMs (red violin plot) compared with untreated BMDMs and that heme exposure made macrophages resistant to the induction of tumoricidal activity by IFN-γ (Figure 6C). Then, an equal number of cells per condition was processed for scRNA-Seq, and a UMAP of 6,808 MC38 tumor cells color-coded for conditions was created (Figure 6D); based on *Ptprc* (CD45) expression, macrophages were excluded from this analysis (Supplemental Figure 4B). Subsequently, we used Leiden clustering to define 3 tumor cell clusters, assigning each cell to a functional state representing proliferation, EMT, or UPR (Figure 6E and Supplemental Figure 4, C and D). In spheroids without macrophages, tumor cells were mainly in the UPR state. Most tumor cells were proliferative in spheroids with untreated BMDMs or IFN-γ–treated BMDMs (Figure 6E). This is a sequela of the tumoricidal macrophage activity, which triggers the compensatory proliferation of the remaining tumor cells. In contrast, in spheroids containing heme-pretreated BMDMs, most tumor cells were in the EMT state, irrespective of IFN-γ treatment.

To integrate the gene expression analysis with the information obtained by the size and fluorescence intensity quantification of the spheroids, we plotted normalized cell densities (Figure 6F) alongside the pathway score intensities for UPR, proliferation, and EMT (Figure 6G). The data visualize that a very high cell density projects on the UMAP area representing EMT in the spheroids containing heme-TAMs, irrespective of whether the macrophages have been treated with IFN-γ. This allowed us to discern that heme abrogates the tumoricidal activity of macrophages, supporting the growth of large cancer spheroids in a persistent EMT state.

We validated our findings in vivo by assessing the metastatic potential of the different spheroids in immunodeficient mice. Three weeks after i.v. injection of an equal number of spheroids per condition and mouse (approximately 750), pulmonary metastases were sparse in mice inoculated with spheroids containing untreated or IFN-γ–treated BMDMs. In contrast, we found extensive metastatic disease in the mice injected with spheroids containing either heme-treated or heme plus IFN-γ–treated BMDMs (Figure 6H and Supplemental Figure 4E). These data confirm that heme exposure undermines the activity of IFN-γ to induce tumoricidal activity in TAMs.

To generalize our findings obtained with MC38 cells, we evaluated the antitumoral activity of heme-pretreated BMDMs using mCherry-4T1 mammary carcinoma cells and GL-261-Luc glioma cells (Supplemental Figure 4F). These tumor cell lines were selected for their representation of different cancer types and their capacity to form spheroids with BMDMs, which was defined in pilot experiments. As with MC38 cells, heme-pretreated BMDMs fostered tumor growth and resistance to IFN-γ with both tumor cell lines. Our data demonstrate that heme exposure in macrophages generates a dominant signal attenuating tumoricidal function and promoting cancer progression.

### Heme signaling progresses via NRF2 to promote heme-TAM transformation, tumor cell growth, invasiveness, and metastasis.

**Figure 7:**
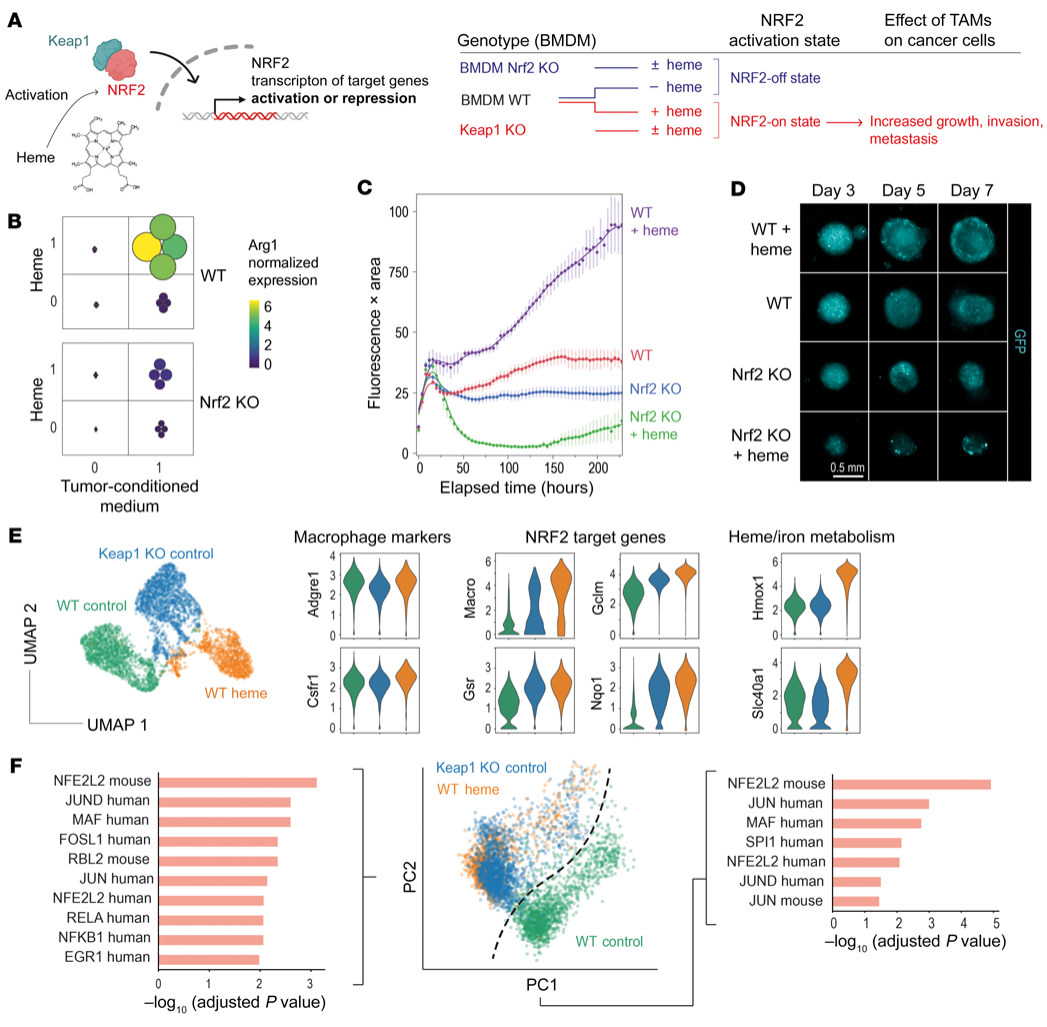
Heme-TAM transformation progresses via NRF2 signaling. (**A**) *Keap1*- and *Nrf2*-KO mice generated BMDMs with locked NRF2-on and NRF2-off states, independent of heme exposure. (**B**) Factorial experiment defining the effect of heme treatment and MC38 cell culture supernatant on the expression of *Arg1* mRNA measured by RT-qPCR in *Nrf2*-WT and *Nrf2*-KO BMDMs. Color and size of the dots indicate the normalized gene expression per sample (*n* = 4 per condition). The data demonstrate that NRF2 is required to leverage the synergistic effect of heme and tumor cell supernatant. (**C**) Live-cell microscopy analysis of spheroids of GFP-MC38 cancer cells mixed with *Nrf2*-KO and *Nrf2*-WT BMDMs that were untreated or pretreated with heme. Data represent the GFP fluorescence intensity integrated across the object area. Data are mean ± 95% CI of 15 replicates analyzed within 1 representative experiment. (**D**) Representative fluorescence images of spheroids. Scale bar: 0.5 mm. (**E**) Multiplexed scRNA-Seq experiment of untreated 2D cultured WT BMDMs, heme-treated WT BMDMs, and untreated *Keap1*-KO BMDMs. The UMAP visualizes that the interaction of genotype and treatment defines distinct gene expression patterns. The violin plots visualize the expression of canonical myeloid marker genes, NRF2-regulated genes, and heme metabolism genes as log_10_ (normalized count + 1) values. (**F**) PCA of the transcriptome data described in **E**. The genes defining PC1 and PC2 were analyzed for driver transcription factors by EnrichR using the TRRUST Transcription Factors 2019 data set. This analysis indicates that heme-treated BMDMs and *Keap1*-KO macrophages share activated NRF2 as a driver of their phenotype.

Activation of NRF2 by heme is a strong antiinflammatory signal in macrophages (27), and the analyses presented above indicate that the expressed transcriptome in heme-TAMs is consistently enriched for NRF2 target genes. Therefore, we delineated the role of NRF2 in the heme-TAM transformation process by performing a series of experiments with *Nrf2*-knockout (*Nrf2*-KO) BMDMs, leading to a locked NRF2-off state, and macrophages with KO of the cytoplasmic NRF2 capture protein KEAP1, leading to a locked NRF2-on state, irrespective of the presence or absence of heme (Figure 7A). We found that *Nrf2* KO interrupted the synergistic effects of heme and MC38 cell–conditioned medium on *Arg1* mRNA expression in 2D BMDM cultures (Figure 7B). In mixed-cell-type spheroids, *Nrf2* KO in macrophages reversed the growth-promoting effect of heme-pretreated BMDMs on MC38 cancer cells. Notably, heme-pretreated *Nrf2*-KO BMDMs had paradoxically enhanced tumoricidal activity, consistent with the unchained proinflammatory function of heme without antiinflammatory NRF2 (Figure 7, C and D). Our findings show that NRF2 signaling is essential for macrophages to acquire the heme-TAM phenotype.

**Figure 8:**
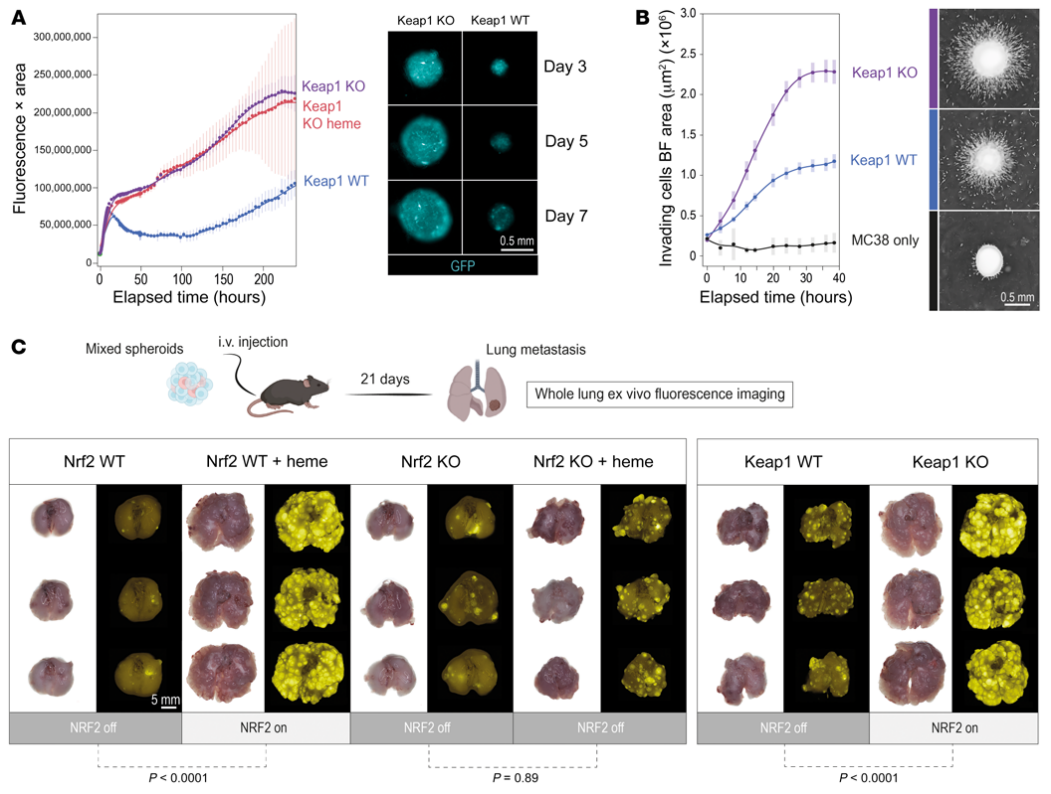
Active NRF2 in TAMs enhances tumor invasiveness and metastatic disease. (**A**) Live-cell microscopy analysis of spheroids of GFP-MC38 cancer cells mixed with untreated WT BMDMs and untreated as well as heme-pretreated *Keap1*-KO BMDMs. Data represent the GFP fluorescence intensity integrated across the object area and demonstrate that *Keap1* KO in macrophages mimics the procancerous effect of heme. Data are mean ± 95% CI of 25 replicates analyzed within 1 representative experiment. GFP fluorescence images of mixed spheroids containing untreated *Keap1*-WT BMDMs and untreated *Keap1*-KO BMDMs on days 3, 5, and 7 after spheroid formation. (**B**) Quantitative spheroid invasion assay. Four days after formation, spheroids were embedded into an extracellular matrix (*t* = 0 hours), and cell invasion was measured with a live-cell imaging system. The invading cell front was automatically segmented and quantified over time. Data are mean ± 95% CI of 20 replicates analyzed within 1 representative experiment. Right: Representative bright-field images of spheroids 16 hours after matrix embedding. Scale bar: 0.5 mm. For better visualization, the images were inverted. (**C**) To test the metastatic potential, approximately 750 spheroids per condition were collected from microwell plates at day 4 after spheroid formation and injected i.v. into *Rag2^−/−^γc^−/−^* mice. Lungs were collected 21 days after injection. Bright-field and GFP fluorescence whole-lung images were used to visualize metastases, which were extensive when NRF2 was active in macrophages. Scale bar: 5 mm. Whole-lung GFP fluorescence intensity was integrated across the lung image area. ANOVA with Tukey-Kramer post-test corrected for multiple comparisons.

Next, to demonstrate that activated NRF2 is sufficient to drive heme-TAM transformation, we analyzed *Keap1*-KO macrophages. In a multiplexed scRNA-Seq experiment comparing the gene expression profiles of untreated wild-type (WT) BMDMs, heme-treated WT BMDMs, and untreated *Keap1*-KO BMDMs, the expression of canonical myeloid markers was similar in both WT and *Keap1*-KO BMDMs, excluding divergent differentiation trajectories of macrophages in response to enhanced NRF2 activity (Figure 7E). However, there were also differences, explaining the divergent UMAP positions of the 3 cell populations. For example, heme-treated BMDMs displayed uniquely high expression of *Hmox1*. In contrast, heme-pretreated WT BMDMs and *Keap1*-KO BMDMs shared increased expression of the NRF2 target genes *Gclm*, *Gsr*, *Slc7a11*, and *Marco*. With the same scRNA-Seq data, we also performed PCA, which is more sensitive for detecting similarities among samples than UMAP-based dimensionality reduction. This analysis revealed that the heme-treated WT BMDMs and the untreated *Keap1*-KO BMDMs were largely overlapping and separate from the untreated WT BMDMs (Figure 7F). The differentially expressed genes defining PC1 and PC2 were highly enriched for NRF2 targets. This confirmed that heme activation of NRF2 is the dominant phenotype driver of heme-TAMs and that constitutive NRF2 activation is sufficient to mimic this phenotype. In line with these gene expression results, we observed that *Keap1*-KO BMDMs fully replicated the growth- and matrix invasion–promoting effects of heme-TAMs in MC38 cancer cell spheroids (Figure 8, A and B).

To validate the procancerous effect of NRF2 activity in TAMs in vivo, we injected GFP-MC38 spheroids containing macrophages with locked NRF2-off or locked NRF2-on i.v. into mice (Figure 8C). We used immunodeficient *Rag2^−/−^γc^−/−^* mice for these studies to avoid interference with endogenous immune responses. Three weeks after i.v. injection of an equal number of spheroids per condition and mouse (approximately 750 spheroids), we observed much more extensive metastatic disease in the mice injected with spheroids containing macrophages with a locked NRF2-on state, irrespective of whether NRF2 was activated by heme or constitutively active as a result of the genetic absence of *Keap1*. In contrast, spheroids containing macrophages with a locked NRF2-off state had low metastatic potential, irrespective of whether the macrophages had been pretreated with heme (Figure 8C and Supplemental Figure 5, A–C).

### Heme NRF2 signaling in hematopoietic cells promotes resistance to immunotherapy.

**Figure 9:**
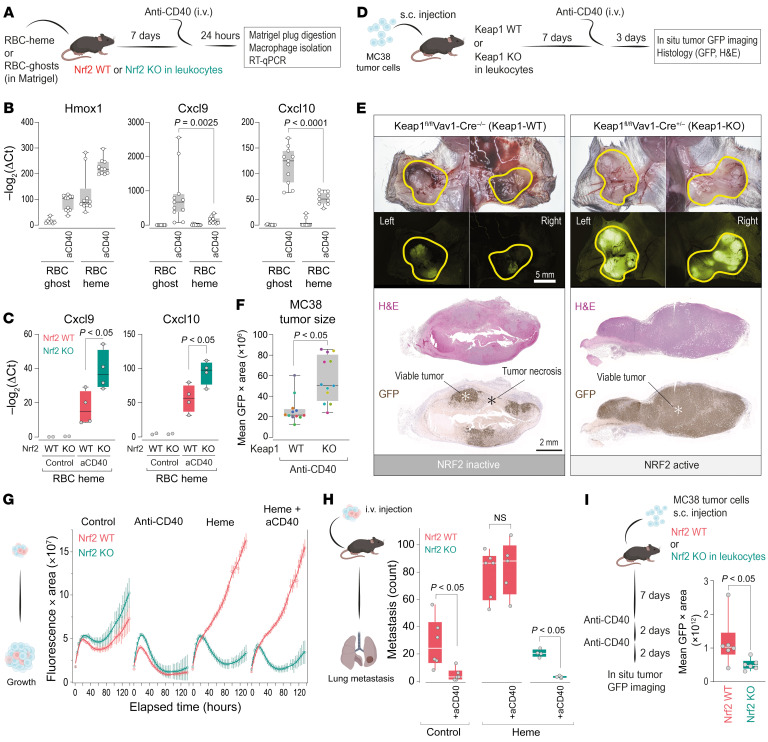
NRF2 signaling in hematopoietic cells promotes resistance to immunotherapy. (**A**) Experimental workflow of Matrigel plug experiments. Macrophages were enriched using F4/80 magnetic beads. (**B**) Relative mRNA expression of *Hmox1*, *Cxcl9*, and *Cxcl10* in macrophages. Each dot represents 1 plug (*n* = 8–11); ANOVA with Tukey-Kramer post-test corrected for multiple comparisons. (**C**) Identical experiments in conditional *Nrf2*-WT and -KO mice. (**D**) Experimental workflow of MC38 tumor cell experiments. (**E**) Top: Bright-field and GFP fluorescence images visualizing MC38 tumors in situ. Scale bar: 5 mm. Bottom: Tumor sections. Scale bar: 2 mm. (**F**) GFP fluorescence intensity integrated across the tumor area. Each dot represents 1 tumor grown on the right and left flank of a mouse; color indicates mouse of origin (*n* = 12); Wilcoxon’s test. (**G**) Live-cell microscopy of spheroids of GFP-MC38 cells mixed with *Nrf2*-KO and *Nrf2*-WT BMDMs that were untreated or pretreated with heme with or without cross-linked anti-CD40 antibody. Data represent GFP fluorescence intensity integrated across object area. Data are mean ± 95% CI of 15 replicates analyzed within 1 representative experiment. (**H**) Spheroids were collected on day 4 after formation and approximately 750 spheroids were injected i.v. per mouse. Lungs were collected after 23 days. Metastatic foci were manually counted on GFP fluorescence whole-lung images. Each dot represents the number of metastatic lesions in 1 mouse (*n* = 4–6). Wilcoxon’s test was used to test for the anti-CD40 antibody effect in the 3 experiments. (**I**) MC38 tumor cells were injected s.c. into conditional *Nrf2-*WT or -KO mice. On day 7, mice were treated with 2 sequential doses of anti-CD40 antibodies, and tumors were imaged 2 days later. Each dot represents 1 tumor (integrated GFP fluorescence intensity across the tumor area, *n* = 6); Wilcoxon’s test.

Finally, we aimed to investigate whether hemorrhage-NRF2 signaling in macrophages could also undermine macrophage reprogramming–based immunotherapy. We first examined whether a hemorrhagic microenvironment changes the transcriptional response of macrophages to agonistic anti-CD40 antibodies. To investigate this, we used our Matrigel scaffold model with RBC-heme or RBC-ghost incorporated (Figure 9A). Seven days after plug placement, we treated the mice with agonistic anti-CD40 antibody. Twenty-four hours later, we detected less expression of *Cxcl9* and *Cxcl10* in F4/80^+^ macrophages of the RBC-heme–enriched plugs, while Hmox1 was superinduced (Figure 9B). The CD40-triggered *Cxcl9* and *Cxcl10* response was significantly more intense when the RBC-heme plugs were placed in conditional *Nrf2*-KO compared with *Nrf2*-WT mice (Figure 9C). This is consistent with the assumption that heme signaling through NRF2 prompted resistance of TAMs to therapeutic reprogramming by anti-CD40 antibodies. To explore the potential therapeutic implications of this finding, we assessed whether active NRF2 within leukocytes of the tumor microenvironment is sufficient to induce resistance to anti-CD40 therapy. We injected GFP-MC38 tumor cells subcutaneously into both flanks of WT mice and those with conditional *Keap1* KO in leukocytes (Figure 9D). After 7 days, the mice were treated with anti-CD40 antibody, and 3 days later, the tumors were recovered for necrosis assessment. On visual inspection of tissue in situ, the tumors in WT animals displayed dark discoloration, providing evidence of extensive tumor necrosis and diminished GFP fluorescence intensity. In contrast, tumors in conditional *Keap1*-KO animals looked viable after treatment with very bright GFP fluorescence (Figure 9E). We also quantified tumor GFP fluorescence intensity after anti-CD40 treatment and found significantly higher fluorescence of the tumors grown in the conditional *Keap1*-KO animals (Figure 9F). These data were substantiated by histology, which revealed extensive tumor necrosis and loss of GFP immunoreactivity in anti-CD40–treated WT mice but not in KO animals (Figure 9E).

To validate the negative interference of heme-NRF2 signaling with CD40-mediated immunotherapy, we performed experiments with *Nrf2*-KO models. Initially, we used our mixed-cell-type spheroid model, which allows us to control heme exposure, NRF2 status, and CD40 activation of macrophages precisely. Live-cell microscopy–based analysis of spheroid growth revealed that antibody-mediated cross-linking of CD40 on macrophages induced vigorous tumoricidal activity, leading to rapid spheroid regression. This effect was not affected by NRF2 status. However, heme pretreatment of WT, not *Nrf2*-KO macrophages, abolished the CD40-induced tumoricidal activity, leading to massive spheroid growth (Figure 9G). These in vitro findings were recapitulated when we injected the spheroids post-formation i.v. into mice. In the first metastasis experiment, we confirmed that CD40 cross-linking on TAMs reduced metastasis formation. A second experiment confirmed that more metastases formed with heme-TAMs and that CD40 cross-linking did not impact metastasis formation in this scenario. In contrast, when we used *Nrf2*-KO macrophages in the third experiment, there was no apparent pro-metastasis effect of heme, and the tumoricidal activity of CD40 cross-linking was restored (Figure 9H).

Finally, we used the subcutaneous MC38 tumor model. We hypothesized that the tumor hemorrhage, which is consistently induced by anti-CD40 therapy, might attenuate the immunotherapeutic response to a second antibody injection by an NRF2-dependent mechanism. Consistent with this hypothesis, we found smaller tumors with barely detectable GFP fluorescence 48 hours after the second antibody injection in mice with a conditional KO of *Nrf2* in leukocytes compared with WT mice (Figure 9I). Collectively, these data suggest that hemorrhage — through heme-activated NRF2 in macrophages of the tumor microenvironment — undermines the anti-CD40 antibody therapeutic response.

## Discussion

Hemorrhage occurs ubiquitously in many solid cancers but has not been considered a biological modifier of cancer biology. Recognizing the quintessential functions of macrophages in the disposal of RBCs, we discovered in this study a trajectory that connects intratumoral hemorrhage to the creation of procancerous TAMs. We found that erythrophagocytosis in the tumor microenvironment initiates this trajectory, elevating intracellular heme levels and activating downstream NRF2 signaling. This prompts a transformative phenotype shift in macrophages, enhancing regenerative capacities while suppressing immune functions. These hemorrhage-derived TAMs drive cancer growth, tissue invasion, and resistance to CD40-agonistic immunotherapy.

The role of macrophage polarization within the tumor microenvironment as a determinant of cancer progression, immune regulation, and treatment outcomes is well established. However, the traditional M1 versus M2 polarization dichotomy has not consistently explained prognostic variability. A comprehensive gene expression analysis across numerous human solid cancer types recently revealed a stronger predictive association with a polarity defined by *CXCL9* and *SPP1* (18). High expression of the IFN-γ response gene *CXCL9* in TAMs corresponded to a favorable prognosis. In contrast, a high *SPP1* and low *CXCL9* expression pattern was associated with poor prognosis and weakened response to immunotherapy. Our regression modeling of cancer mRNA expression data from over 11,000 patients in the TCGA PANCAN database suggested that heme exposure, measured by *HMOX1* expression, strongly predicts *SPP1*. In line with this finding, our experiments showed that heme-NRF2 signaling reproduced the *Spp1*^hi^*Cxcl9*^lo^ poor-prognosis phenotype in mouse BMDMs, reinforcing that hemorrhage might be a universal and clinically important TAM polarization factor. Thus, systematic studies are warranted to explore the relationship between spontaneous or treatment-induced hemorrhage, tumor microenvironmental heme exposure, and disease outcomes in various cancers.

Heme-activated NRF2 endows TAMs with an immunosuppressive phenotype, promoting cancer cell EMT, matrix invasion, and high metastatic potential while impairing tumoricidal activity. These effects of NRF2 activation in TAMs involve a combination of intrinsic and extrinsic mechanisms. Intrinsic mechanisms lock TAMs into an immune-suppressed state, providing resistance against macrophage activation induced by IFN-γ and agonistic anti-CD40 antibodies. In contrast, extrinsic mechanisms offer anti-apoptotic and pro-invasion signals to cancer cells. Collectively, it is likely that NRF2-educated TAMs adversely affect outcomes of solid cancers. Further investigation has to establish the molecular nature of the mechanisms transforming macrophage functions downstream of NRF2.

Tumor cell–intrinsic NRF2 has been comprehensively studied and is known to play dual roles in tumorigenesis, acting as both a tumor suppressor and promoter, depending on the context and disease stage (37–39). NRF2 primarily supports redox homeostasis by activating genes involved in antioxidant enzyme production and detoxification proteins, offering a safeguard against cancer development (40–43). However, persistent NRF2 activation in established cancer cells, often resulting from mutations in *KEAP1* or *NRF2*, promotes tumor cell survival, growth, and resistance to therapy (44, 45). Less is known about the regulators of NRF2 activity in cells of the cancer microenvironment and how NRF2 activation there may feed back on cancer progression and anticancer immune responses. NRF2 induces extensive transcriptional and metabolic reprogramming in myeloid cells (46). Beyond attenuating intrinsic tumoricidal functions, as our studies show, elevated NRF2 activity in macrophages shapes an immunosuppressive milieu that impairs cytotoxic T cell functionality and fosters immune tolerance through Treg recruitment (47, 48). Concurrently, NRF2 activation modulates extracellular matrix composition (49), potentially promoting tumor invasiveness. Furthermore, heme exposure and NRF2 activation intensify glycolysis and lactate production (50–53), possibly underpinning a harmful metabolic coupling between TAMs and cancer cells (54–56).

Given its chemistry, the oxidant heme has the potential to broadly activate NRF2 across cell types and tissues (57). However, physiological mechanisms, such as hemoglobin coordination and packaging within the RBC membrane, RBC antioxidant metabolites, and extracellular scavenger proteins, effectively shield tissue environments against oxidative impact from heme (31, 57, 58). Instead, the hemorrhage-triggered wound healing response recruits macrophages (59), which have the unique ability to recognize and ingest damaged RBCs and heme-protein complexes (60), allowing them to shuttle heme toward a system capable of safely degrading and reutilizing its components (61). In this context, NRF2 activation forms part of the adaptive response of macrophages (27, 62–65). Thus, RBC-heme may be considered a uniquely macrophage-specific NRF2 activator in the hemorrhagic tumor microenvironment.

Our findings also align with previous observations linking HMOX1-expressing macrophages in the tumor microenvironment to cancer progression and unfavorable disease outcomes (66–68). Notably, our findings connect this association to a pathophysiological pathway initiated by spontaneous or therapy-induced intratumoral hemorrhage. HMOX1 is most strongly induced in macrophages among heme-induced genes and is, therefore, a perfect marker for this process. Nevertheless, in our studies, NRF2 activation was sufficient to transmit TAM reprogramming, with no apparent functional role of the marker HMOX1 in this process. We learned to interpret studies of HMOX1 inhibition and knockout models very cautiously. In the absence of HMOX1, cells are exceedingly vulnerable when exposed to heme, and some effects may reflect the consequences of cell depletion in heme-rich microenvironments rather than more specific signaling functions of the enzyme or its downstream metabolites (57).

Overall, our discoveries potentially explain the limited effectiveness of conventional therapies and immunotherapies in overtly hemorrhagic tumors, such as gliomas (69), and the progressive immunoresistance observed across various malignancies (70). Further research could develop novel therapies that harness NRF2 signaling in macrophages to boost cancer immunity and overcome treatment resistance.

## Methods

Additional and detailed protocols and reagents are provided in Supplemental Methods and Supplemental Tables 1 and 2.

### Mouse strains and breeding

C57BL/6J (JAX strain) mice were obtained from Charles River Laboratories. To generate *tdTomato^+^* macrophages, *Vav-Cre* mice, obtained from the Swiss Immunological Mouse Repository (SwImMR), were bred with *Ai14^tdTomato^* mice (The Jackson Laboratory). To generate conditional *Keap1*-KO mice, *Keap1^tm2.Mym^* (71) mice were obtained from RIKEN BioResource Research Center and crossed with *Vav-Cre* mice. To generate conditional *Nrf2*-KO mice, C57BL/6-*Nfe2l2tm1.1Sred/SbisJ* (*Nrf2^flox^*) (72, 73) mice were obtained from The Jackson Laboratory and crossed with *Vav-Cre* mice. Control littermates without the Cre driver were used for experiments involving these mouse strains. *Nrf2*-KO and -WT littermates were obtained from Yuet Wai Kan (University of California, San Francisco, California, USA). *Rag2^−/−^γc^−/−^* mice were obtained from SwImMR. For all studies, mice were randomly allocated to treatment groups, and the investigators were blinded to allocation during experiments and outcome assessment.

### Cell lines and primary BMDM cultures

Methods for tumor cell and BMDM cultures are provided in Supplemental Methods.

### Heme preparation for cell culture

Hemin (heme-chloride) was obtained from Frontier Scientific (Newark). Batches were tested endotoxin-free and prepared as heme-albumin for cell treatments as previously described (60).

### 3D tumor spheroid production, culture, and analysis

#### Single-spheroid culture.

5 × 10^3^ GFP-MC38 cells, 2.5 × 10^3^ mCherry-4T1 cells, or 5 × 10^3^ GL261-Luc cells with or without BMDMs (at a 1:1 ratio) were seeded in 100 μL tumor cell culture medium in 96-well Ultra-low Attachment Plate PrimeSurface 3D Culture Spheroid plates (S-BIO). M-CSF (100 ng/mL) was added to all spheroid cultures irrespective of the addition of BMDMs to control for the cytokine effect. For live-cell apoptosis imaging, Annexin V red (Sartorius) was added to the medium, as instructed by the manufacturer.

#### Multispheroid culture in microwell plates.

GFP-MC38 cells (5 × 10^4^) with or without BMDMs (at a 1:1 ratio) were seeded in 800 μL of tumor cell medium with M-CSF (100 ng/mL) in a 24-well SphericalPlate 5D microwell (Axonlab). Eight hundred microliters of fresh culture cell medium was added on day 3.

#### Quantification of spheroid growth and invasion.

Single spheroids were imaged with an IncuCyte S3 instrument (Sartorius). The area and fluorescence intensities of the images were measured using the IncuCyte Spheroid Software Module (Sartorius). Data are reported as spheroid fluorescence intensity integrated across the spheroid area (for tumor cells expressing a fluorescent protein) or as spheroid area. For the spheroid invasion assay, a mask based on the invading cell area was created automatically with the IncuCyte Spheroid Software Module. Multispheroids were scanned using a Zeiss Axio Observer Z1 microscope. The spheroid area was quantified manually in QuPath (74) (v0.3.0), and fluorescence intensity was measured using QuPath’s intensity feature plug-in. The spheroids were detected in the EGFP/FITC channel using a set pixel size of 1.26 μm.

### Mouse models

#### Subcutaneous Matrigel plug model in mice.

Matrigel mixture (350 μL) was injected subcutaneously into the flanks of anesthetized (intraperitoneal injection of 80 mg/kg ketamine, 16 mg/kg xylazine, 3 mg/kg acepromazine) C57BL/6J mice using a 24-G needle. After 7 days, mice were euthanized, and plugs were removed for downstream analysis. For anti-CD40 antibody experiments, the mice were treated i.v. on day 7 with an agonistic anti-CD40 antibody (20 mg/kg; InVivoPlus, Bio X Cell clone FGK4.5) or an isotype control antibody.

#### Lung metastasis model in mice.

Approximately 750 spheroids were collected from microwell plates (equal to the content of 1 macro well) and injected i.v. into the tail vein of C57BL/6J or *Rag2^−/−^γc^−/−^* mice. Three weeks after injection, the lungs of anesthetized mice were perfused with PBS through the right ventricle and the trachea and collected for whole-organ fluorescence imaging with a Zeiss Discovery V8 stereomicroscope and histology. For the metastasis experiments shown in Figure 9H, metastases were manually counted to enhance sensitivity and specificity in the low disease burden range to assess the effects of anti-CD40 treatment.

#### Tumor growth model in mice.

Once confluent, GFP-MC38 tumor cells were harvested using 5 mM EDTA (Gibco) (4 minutes at 37°C) and washed twice in PBS. MC38 cells (2 × 10^6^) in culture medium were mixed with Geltrex (Thermo Fisher Scientific) and injected subcutaneously into the mouse flanks. Agonistic anti-CD40 treatment (20 mg/kg; InVivoPlus, clone FGK4.5) or an isotype control antibody was administered i.v. as indicated. Mice were euthanized, and tumors were collected 2 or 3 days after antibody administration. GFP fluorescence was measured immediately, and tumors were then fixed in formalin (10%) and stored at room temperature.

### Sequencing-based workflows and data analysis

We used the 10x Genomics workflows for scRNA-Seq (Chromium Next GEM Single Cell 3′ v3.1) and spatial RNA-Seq (Visium CytAssist Spatial Gene Expression for FFPE). Ready-made libraries were sequenced at the Functional Genomics Center Zurich on an Illumina NovaSeq 6000 system. Downstream analysis was performed in Python (version 3.8.6) with Scanpy (1.7.0) (75). Details of sample preparation and data analysis are provided on an experiment-by-experiment basis in Supplemental Methods.

### Statistics

Data plotting and statistical analysis were performed with Prism 9 (GraphPad) and JMP 15 (SAS). We used 1-way ANOVA with Tukey-Kramer post-test to account for multiple comparisons and *t* tests (2-tailed) or Wilcoxon’s tests, as indicated in the figure legends. *P* values of less than 0.05 were considered significant. All data points are displayed in box plots to visualize the data distribution. Analysis of sequencing data is described in Supplemental Methods.

### Study approval

All animal experiments were performed according to animal experimentation licenses as approved by the Swiss Federal Veterinary Office.

### Data availability

Sequencing data are publicly available (Gene Expression Omnibus accession GSE237612). Detailed information is provided on an experiment-by-experiment basis in Supplemental Methods. A Supporting Data Values file is provided in Supplemental Methods.

## Author contributions

DJS designed the study, performed experiments, analyzed the data, and wrote the manuscript. NSL performed the mouse experiments and analyzed the data. LB performed the RNA-Seq experiments and analyzed the data. KH performed the histological experiments. RMB performed experiments, analyzed data, and reviewed the manuscript. RH acquired the animal experimentation licenses, performed confocal microscopy, and designed the graphical abstract. ED analyzed the sequencing data. FV designed the study, performed experiments, analyzed data, and wrote the manuscript.

## Supplementary Material

Supplemental data

Supporting data values

## Version 1 12/07/2023

In-Press Preview

## Version 2 02/01/2024

Electronic publication
