# Supplementary material for: Hemorrhage-activated NRF2 in tumor-associated macrophages drives cancer growth, invasion, and immunotherapy resistance
Source: J Clin Invest. 2024 Feb 1;134(3):e174528. doi: 10.1172/JCI174528 (PMC10849758; doi:10.1172/JCI174528)
Supplement: Supplemental data [file jci-134-174528-s010.pdf]

Hemorrhage-activated NRF2 in tumor-associated macrophages drives cancer growth, invasion, and immunotherapy resistance

Dominik J. Schaer[1], Nadja Schulthess-Lutz[1], Livio Baselgia[1], Kerstin Hansen[1], Raphael M. Buzzi[1], Rok Humar[1], Elena Dürst[1], Florence Vallelian*[1]

[1] Department of General Internal Medicine, University Hospital and University of Zurich, Zurich, Switzerland

*Correspondence: florence.vallelian@usz.ch

Page 2 - 6: Supplementary Figures 1 - 5

Page 7 - 26: Supplementary Methods

Supplementary Figure 1

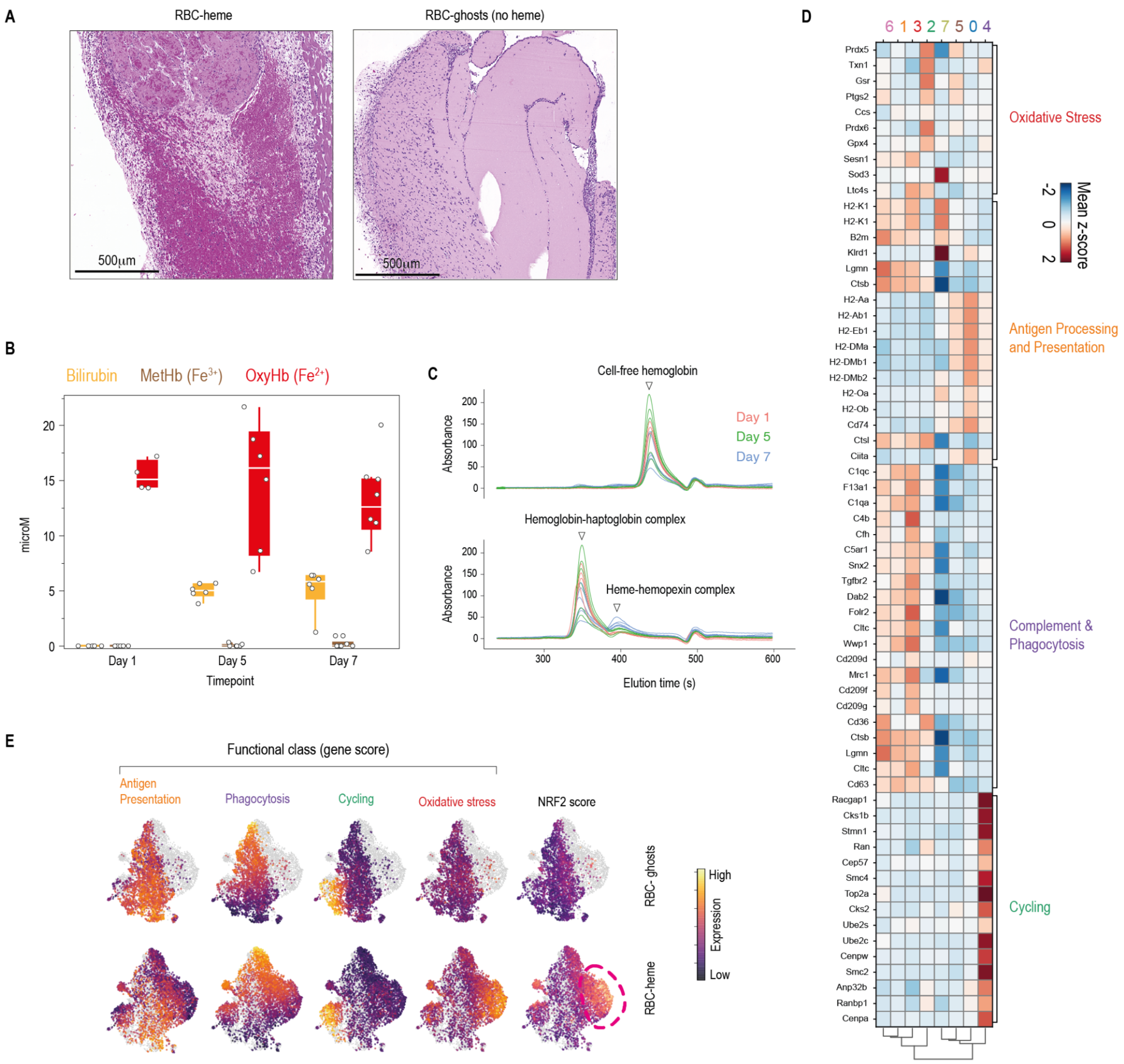

Supplementary Figure 1 (related to Figure 2 and Figure 3)

A. Representative Matrigel plug paraffin sections stained with H&E. Scale bar = 500 µm.
B. Spectral deconvolution of absorption spectra from digested Matrigel scaffolds reveal a consistent concentration of oxyhemoglobin and increased amounts of bilirubin in plugs removed on day 5 or 7 compared to plugs removed on day 1.
C. Size-exclusion chromatography elution profiles at 414 nm of digested Matrigel plugs measured directly after digestion and after adding haptoglobin and hemopexin. Most hemoglobin is complexed within the added haptoglobin. In the day 7 samples, a small amount of free heme binds to hemopexin and appears as a heme-hemopexin complex.
D. Expression heatmap of macrophage functional class-associated genes in each cluster from merged scRNA-seq of RBC-heme and RBC-ghost plugs. Data are standardized by row, and Z scores are displayed by color code.
F. Scored expression of the functional classification gene sets and the NRF2 score (TRRUST). The dashed line highlights the oxidative stress cluster in the RBC-heme plug, as highlighted in Figure 3D.

Supplementary Figure 2

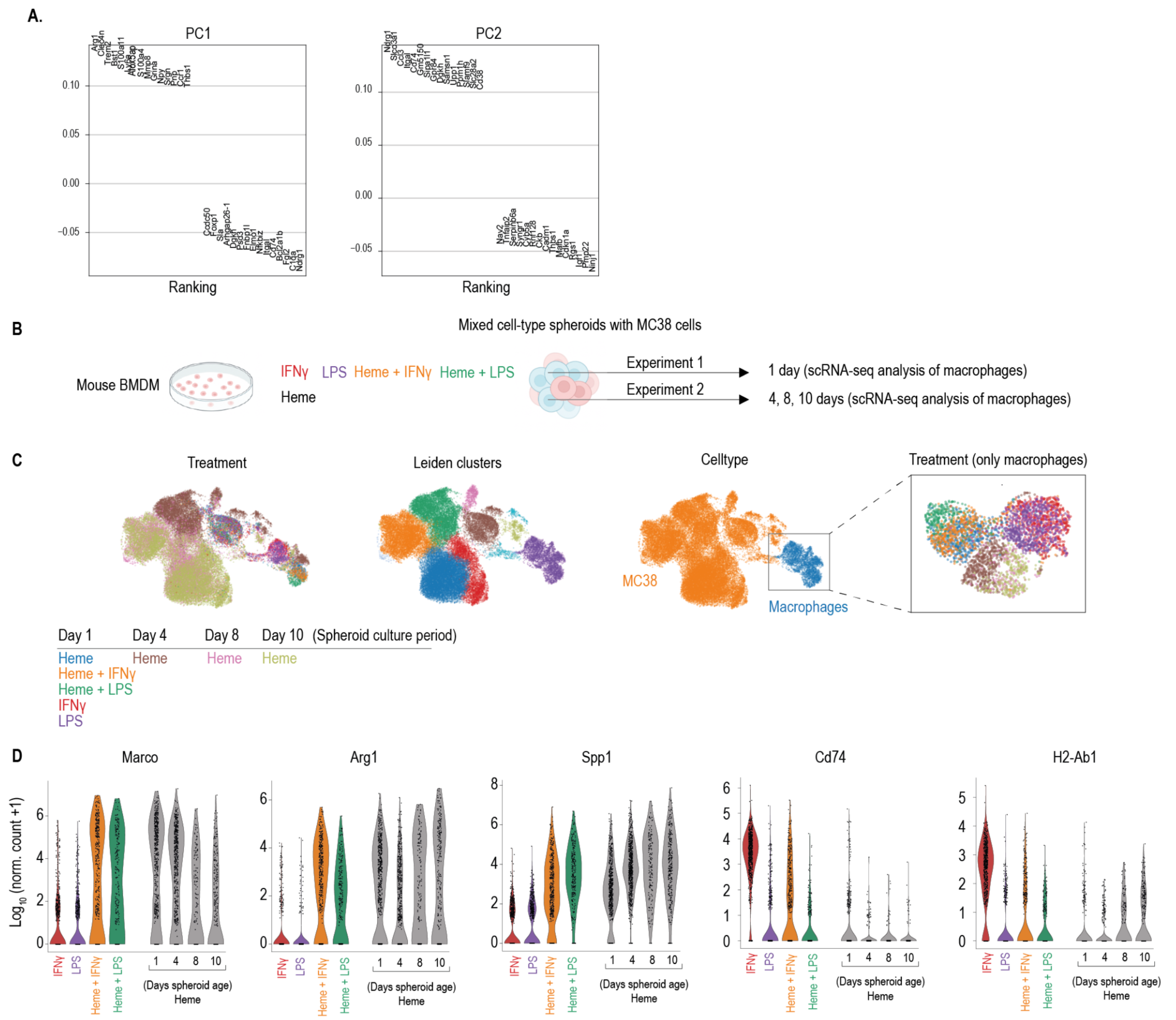

Supplementary Figure 2 (related to Figure 4 and Figure 5)

A. PCA of spheroid macrophages. Top positive and negative driving genes of PC1 and PC2.
B. Workflow for 3D-spheroid cell culture experiments.
C. scRNA-seq count data from the pooled experiment described in Figure 4F and Figure 5D. UMAPs are color-coded for Leiden clustering, cell type, and treatment. Macrophages were extracted, and a novel UMAP was calculated.
D. Expression of selected marker genes in macrophages of mixed cell-type spheroids grown for different periods after cell mixing and spheroid formation. scRNA-seq count data were extracted from the respective datasets, pooled, batch-corrected, and visualized as violin plots. Data for IFNγ, LPS, heme + IFNγ, heme + LPS, and heme on day 1 are from the experiment described in Figure 4F. Data from days 4, 8, and 10 were extracted from the experiment described in Figure 5D.

Supplementary Figure 3

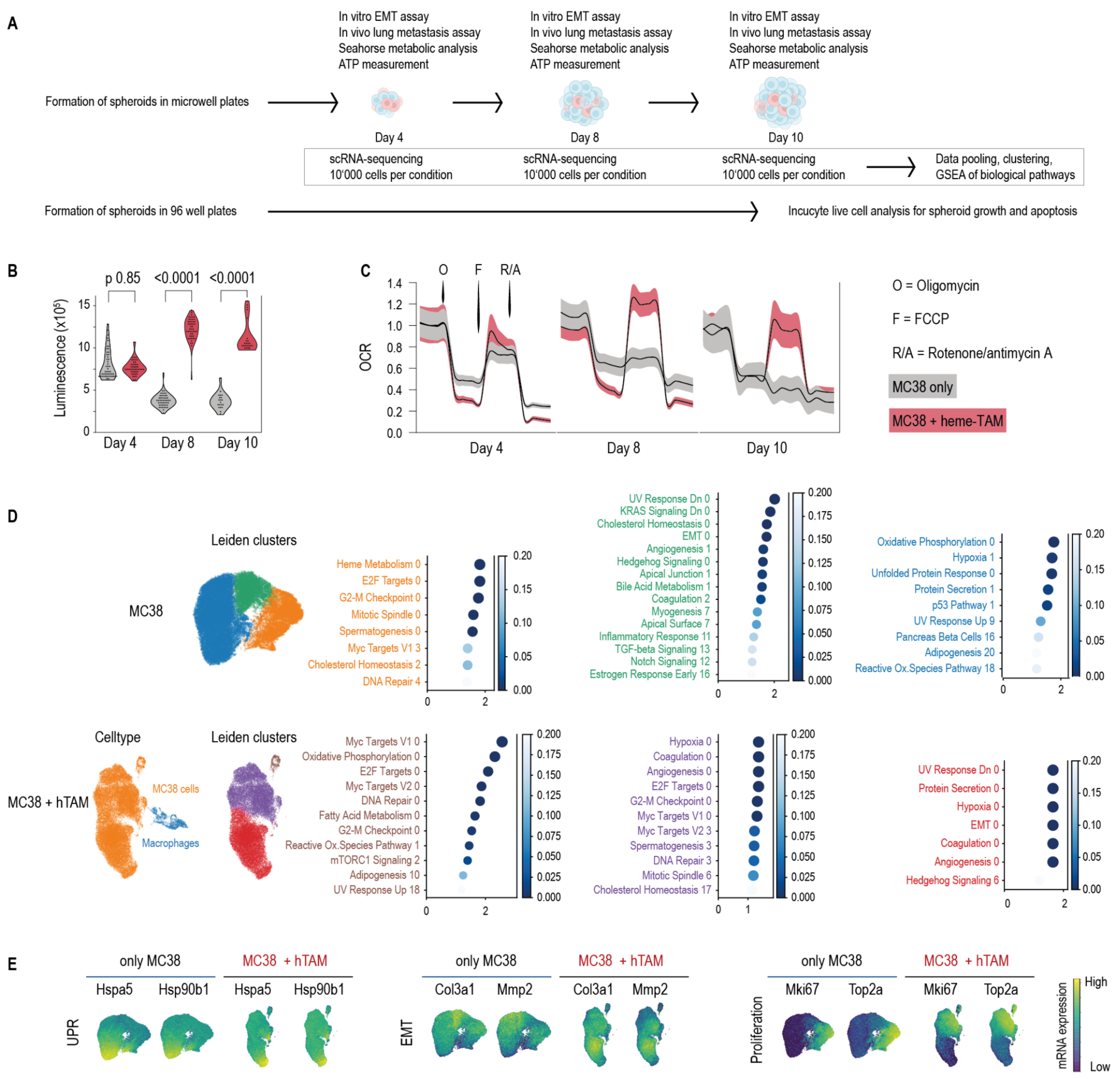

Supplementary Figure 3 (related to Figure 5)

A. Workflow for 3D-spheroid cell culture experiments.

B. ATP was measured in single spheroids cultured in 96-well plates on days 4, 8, and 10 with a luminescence assay (gray = MC38, red = MC38 + heme-TAMs) (n = 24-48). Each dot represents one spheroid (t-test).

C. Oxygen consumption rate (OCR) of spheroids was measured on days 4, 8, and 10 at baseline and after sequential addition of oligomycin (O), FCCP (F), and rotenone/antimycin (R/A). Data are the mean ± 95% CI from 5 replicates per condition (gray = MC38 spheroids, red = MC38 + heme-TAMs).

D. Multiplexed MC38 tumor cell spheroids or mixed spheroids (MC38 + heme-TAMs) for scRNA-seq experiments were collected on days 4, 8, and 10 post-spheroid formation. Macrophages were excluded for further analysis. UMAPs are color-coded for cell type (MC38 + heme-TAMs) and Leiden clustering, defining three clusters per experiment. Clusters were functionally annotated according to GSEA.

E. Expression of selected signature genes of the three functional GSEA categories (UPR: unfolded protein response; EMT: epithelial-mesenchymal transition).

Supplementary Figure 4

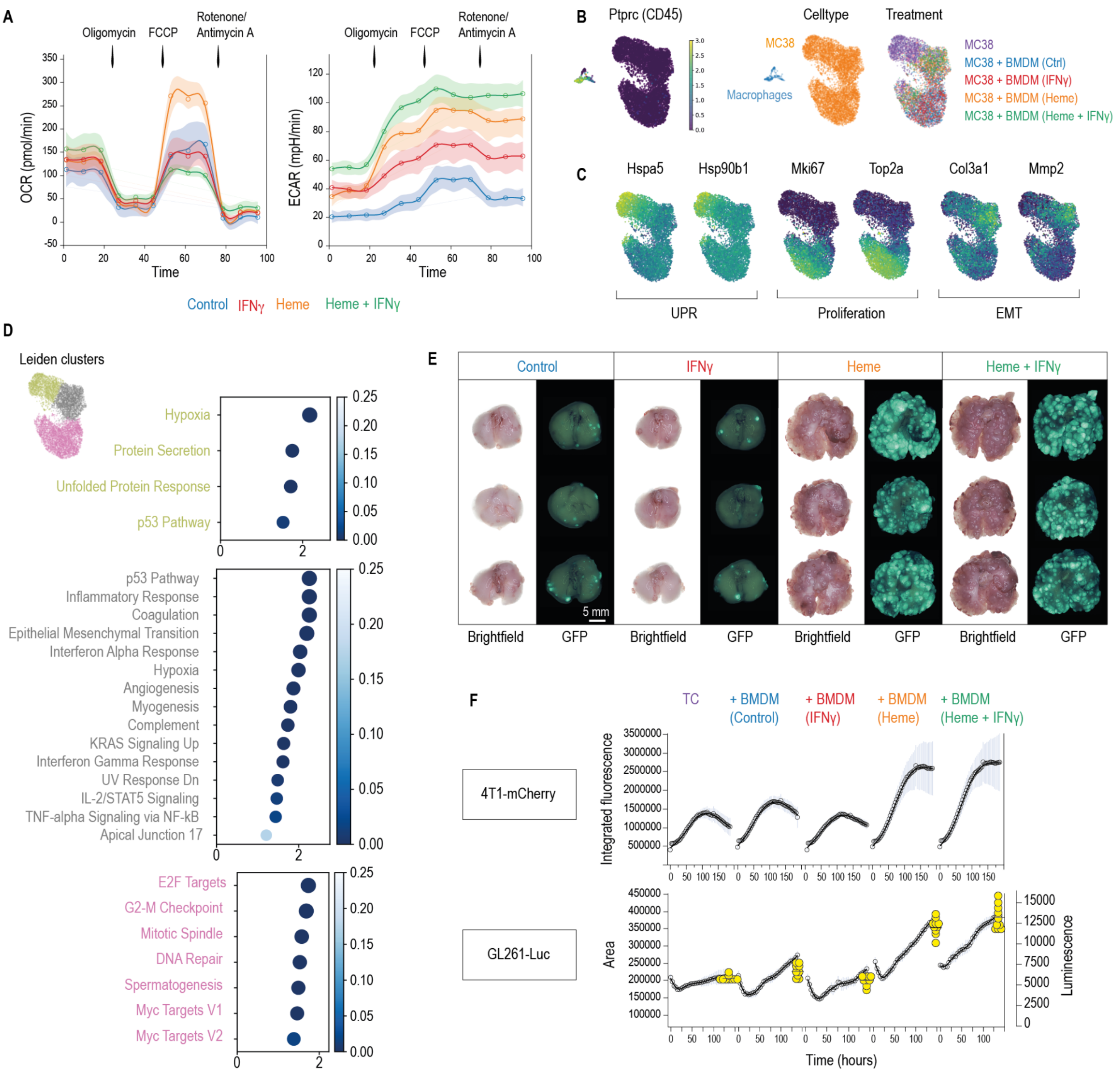

Supplementary Figure 4 (related to Figure 6)

A. Oxygen consumption rate (OCR) and acidification rate (ECAR) of control, IFNγ, heme or heme + IFNγ-treated BMDMs was measured at baseline and after sequential addition of oligomycin (O), FCCP (F), and rotenone/antimycin (R/A). Data are the mean ± 95% CI from five replicates per condition.
B. Multiplexed scRNA-seq analysis of cell-type spheroids collected on day 9. UMAPs are color-coded for Ptprc (CD45), cell type, treatment (after macrophage exclusion), and Leiden clustering.
C. The three clusters were functionally annotated according to GSEA.
D. Expression of selected signature genes of the three functional GSEA categories
E. Approximately 750 mixed cell-type spheroids (GFP-MC38 cancer cells + BMDMs) were collected from microwell plates on day 4 post-formation and injected i.v. into *Rag2*$^{-/-}$*γc*$^{-/-}$ mice. Lungs were harvested 20 days after injection. Brightfield and GFP fluorescence images depict the extent of metastatic disease. Scale bar = 5 mm.
F. Live cell microscopy of mCherry-4T1 and GL261-Luc cancer cell spheroids and mixed spheroids of

these cancer cells with differentially pretreated BMDMs. For mCherry-4T1 spheroids, data are plotted as the mean ± 95% CI (n=10) of the red fluorescence integrated across the spheroid area. For GL261-Luc spheroids, data are plotted as the mean ± 95% CI (n= 10) of the spheroid area, and luminescence of each spheroid was measured at the end of the study after adding D-luciferin to the cell culture medium (ANOVA with Tukey–Kramer posttest corrected for each comparison, GL261-L vs. BMDMs IFNγ p 0.97, GL261-L vs. control BMDMs p 0.30, control BMDMs vs. IFNγ BMDMs p 0.10, heme BMDMs vs. heme + IFNγ p 0.31, further comparisons p< 0.0001).

Supplementary Figure 5

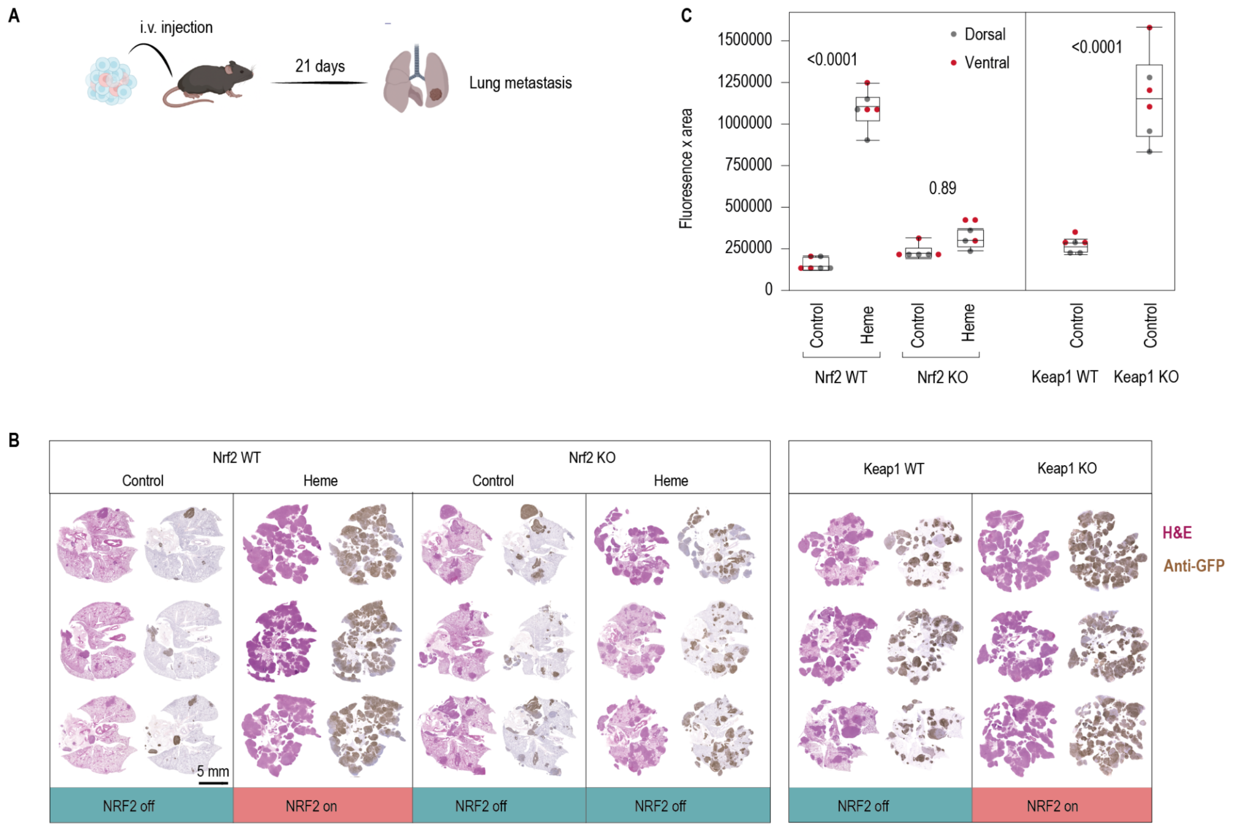

Supplementary Figure 5 (related to Figure 8)

A. Experimental workflow of lung metastasis model

B. Approximately 750 mixed spheroids per condition were collected from microwell plates on day 4 post-spheroid formation and injected i.v. into *Rag2*$^{-/-}$*γc*$^{-/-}$ mice. Lungs were harvested 21 days after injection. Representative lung paraffin sections were stained with H&E and for GFP. Scale bar = 5 mm.

C. Whole-lung GFP fluorescence intensity was integrated across the lung image area (gray dots= dorsal view, red dots = ventral view). ANOVA with Tukey–Kramer posttest corrected for multiple comparisons.

Hemorrhage-activated NRF2 in tumor-associated macrophages drives cancer growth, invasion, and immunotherapy resistance

Dominik J. Schaer, Nadja Schulthess-Lutz, Livio Baselgia, Kerstin Hansen, Raphael M. Buzzi, Rok Humar, Elena Dürst, Florence Vallelian

Supplementary Methods - Detailed procedures and protocols

Data and code availability

Sequencing data are publicly available (GEO accession code GSE237612).

Experiment	Type of data	GEO Accession	GEO Samples	Reviewer Token
Fig.1 (Spatial tumor)	spRNA-seq	GSE237119	GSM7595667, GSM7595668	ixwjuicqfnaxtql
Fig.3 (Matrigel plugs)	scRNA-seq	GSE236439	GSM7544071, GSM7544072	crmxygwubfydten
Fig.4 (Spheroids day 1)	scRNA-seq	GSE236576	GSM7558128, GSM7558129, GSM7558130, GSM7558132, GSM7558133	gnspqmsovnwlfkt
Fig.4A/B (BMDM bulk)	Bulk RNA-seq	GSE237238	GSM7597600, GSM7597601, GSM7597602, GSM7597603, GSM7597604, GSM7597605	gdcpamyovhslhmt
Suppl.Figure 2	scRNA-seq	GSE237257		
Fig.5 (Spheroids day 4, 8, 10)	scRNA-seq	GSE236995	GSM7592776, GSM7592777, GSM7592778, GSM7592779, GSM7592780, GSM7592781	mdyfygyghxovnwz
Fig.6 (Spheroids day 9)	scRNA-seq	GSE236996	GSM7592782, GSM7592783, GSM7592784, GSM7592785, GSM7592786	qbmdoukkxfknlkv
Fig.7 (BMDM 2D)	scRNA-seq	GSE236997	GSM7592788. GSM7592789. GSM7592790	kjalwoikxnkhvaf
**All of the above**		**GSE237612**		exqdcmwknjonpmx

TCGA data analysis - Regression modeling

We used Xena browser (1) to explore data in The Cancer Genome Atlas (TCGA) Pan-Cancer (PANCAN) database (https://www.cancer.gov/tcga) and to plot survival curves for *CD163* and *SPP1* expression, including all solid cancer types excluding lymphoma and leukemia. The log2(count+1)-transformed and batch-corrected gene expression data were exported from the database and further analyzed in JMP 15 (SAS Institute Inc.). To make sure that the expression data of our marker genes were not excessively zero-inflated, we confirmed approximately normal distribution of the log2(count+1)-transformed data using the `Distribution` function combined with `Fit normal` and `Normal quantile plot` functions before computing multiple linear regression models (ordinary least squares method) using CD163 and SPP1 as the

response variables and the tissue microenvironmental factors as predictors. In Figures 1A and 1B, we report the overall fit of the model (r^2) and the coefficient for each predictor gene with the respective p-value.

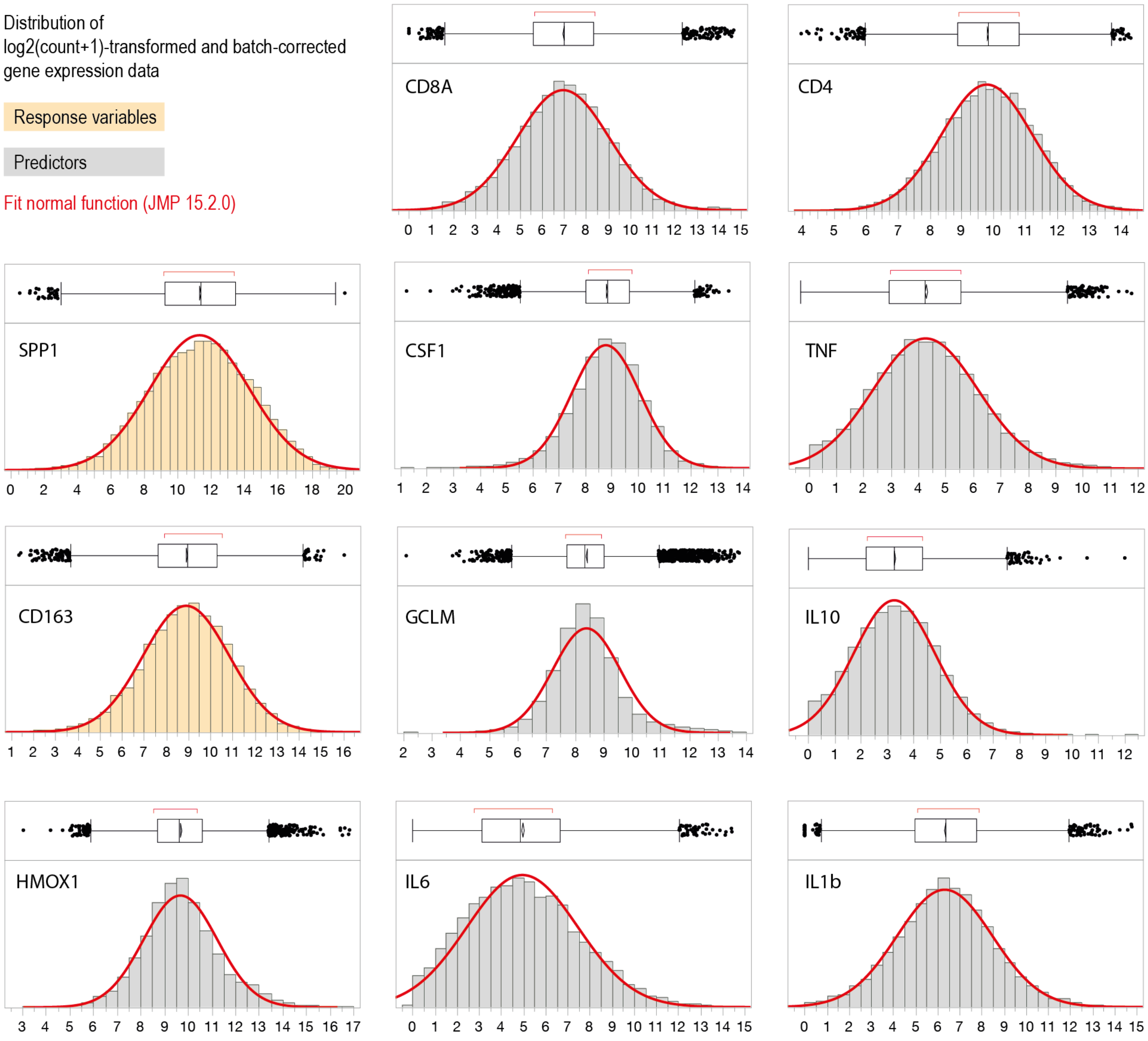

Animals

C57BL/6J (JAX^{TM} strain) mice were obtained from Charles River Laboratories. To generate tdTomato^{+} macrophages, Vav-Cre mice, obtained from the Swiss Immunological Mouse repository (SwImMR), were bred with *Ai14*^{tdTomato} mice (The Jackson Laboratory). *Conditional Keap1 knockout mice: Keap1*$^{tm2.Mym}$ (2) mice were obtained from RIKEN BRC and crossed with Vav-Cre mice. *Conditional Nrf2 knockout mice:* C57BL/6-*Nfe2l2tm1.1Sred/SbisJ* (*Nrf2*^{flox}) (3, 4)

mice were obtained from Jackson laboratories and crossed with *Vav-Cre* mice. Control littermates without the Cre driver were used for experiments involving these mouse strains. *Nrf2*$^{-/-}$ and WT littermates were obtained from Professor Yuet Wai Kan (University of California, San Francisco). *Rag2*$^{-/-}$*γc*$^{-/-}$ mice were obtained from the SwImMR. All breeding colonies were housed and bred in the specific pathogen-free (SPF) animal facility at the Laboratory Animal Services Center (LASC) of the University of Zurich in individually ventilated cages. Males and females mice aged 7-12 weeks were used for all experiments, and all experiments with mice were performed according to animal experimentation licenses approved by the Swiss Federal Veterinary Office. For all studies, mice were randomly allocated to treatment groups, and the investigators were blinded to allocation during experiments and outcome assessment.

Cell lines and primary cultures

Tumor cell line culture

GFP-MC38 (donated by Gerhard Christofor, Department of Biomedicine, University of Basel, Basel, Switzerland) were cultured in RPMI-1640 medium supplemented with 10% fetal bovine serum (FBS, Gibco), 1% penicillin/streptomycin (P/S, Gibco), 1% nonessential amino acids (NEAA, Gibco) and 1% sodium pyruvate (Gibco). A cell line with homogeneous GFP expression was obtained by FACS sorting. Cell line authentication was performed before and after cell sorting by Short Tandem Repeat (STR) DNA genotype analysis (Microsynth, Balgach, Switzerland). mCherry-4T1 (donated by Lubor Borsig, Institute of Physiology, University of Zurich, Zurich, Switzerland) were cultured in Dulbecco's Modified Eagle's Medium (DMEM, Gibco) supplemented with 10% FBS and 1% P/S. GL-261-Luc cells (donated by J. vom Berg, Institute for Laboratory Animal Science, University of Zurich) were cultured in DMEM supplemented with 10% FBS and 1% P/S. Cell cultures were maintained at 37°C and 5% CO_2 in a humidified incubator. All cells used in this study were confirmed to be negative for mycoplasma.

BMDM culture

BM cells were isolated by flushing the femurs and tibias of 7- to 12-week-old C57BL/6J mice and then passed through a 70-µm filter. The BM cells were plated at a density of 3 x 10^5 cells/ml on tissue culture-treated 60 mm UpCell dishes (Nunc™ UpCell™, ThermoFisher) in complete RPMI-1640 medium (10% fetal calf serum (FCS), 1% L-glutamine and 1% P/S) supplemented with 100 ng/ml recombinant mouse M-CSF (PeproTech). On day 3, half of the medium was replaced.

Cultures were treated on day 3 with 300 µM heme. For inflammatory polarization, IFNγ (10 ng/ml, PeproTech) or LPS (10 ng/ml, Sigma) was added on day 6 for 24 hours. For in vitro anti-CD40 stimulation, FGK45 (1 µgr/ml, InVivoPLus) was crosslinked with goat anti-rat immunoglobulin G (0.5 µgr/ml, BioLegend) for 30 min at room temperature before addition to the culture medium for 24 hours. The BM cells were harvested for analysis on day 7 from the temperature-responsive cell culture plates after cooling to room temperature. Cells were washed twice in PBS and centrifuged (300*g*, 10 min) before processing. For experiments involving conditioned medium from tumor cells, BMDMs were seeded after washing at the end of the differentiation period in 12-well plates (TPP) in MC38 conditioned medium overnight and lysed in RNA lysis buffer 1% β-mercaptoethanol for transcriptome analysis.

Heme preparation for cell culture

Hemin (heme-chloride) was obtained from Frontier Scientific (Newark). Batches were tested endotoxin-free and prepared as heme-albumin for cell treatments as described (5).

3D tumor spheroid production, culture, and analysis

Single-spheroid culture

5×10^3 GFP-MC38 cells, 2.5×10^3 mCherry-4T1 cells, or 5×10^3 GL261-Luc cells ± BMDMs (at a 1:1 ratio) were seeded in 100 µl tumor cell culture medium in 96-well Ultralow Attachment Plate PrimeSurface® 3D Culture Spheroid plates (S-BIO). M-CSF (100 ng/ml) was added to all spheroid cultures irrespective of the addition of BMDMs to control for the cytokine effect. For live cell apoptosis imaging, Annexin V red (Sartorius) was added to the medium, as instructed by the manufacturer. For spheroid invasion assays, 100 µl of Cultrex Spheroid Invasion Extracellular Matrix (Bio-Techne) was added on day 4.

Multispheroid culture in microwell plates

GFP-MC38 cells (5×10^4) ± BMDMs (at a 1:1 ratio) were seeded in 800 µl of tumor cell medium with M-CSF (100 ng/ml) in a 24-well SphericalPlate® 5D microwell (Axonlab). 800 µl of fresh culture cell medium was added on day 3.

Quantification of spheroid growth and invasion

Single spheroids were imaged in the cell culture incubator with an IncuCyte S3 instrument (Sartorius). Green or red fluorescence and phase contrast images of the spheroids were acquired

every 4 hours for seven to ten days. The area and fluorescence intensities of the images were measured using the IncuCyte Spheroid Software Module (Sartorius). Data are reported as spheroid fluorescence intensity integrated across the spheroid area (for tumor cells expressing a fluorescent protein) or as spheroid area. For the spheroid invasion assay, a mask based on the invading cell area was created automatically with the IncuCyte Spheroid Software Module. Multispheroids were scanned using a Zeiss Axio Observer Z1 microscope. The spheroid area was quantified manually in QuPath (6) (v0.3.0), and fluorescence intensity was measured using QuPath's intensity feature plugin. The spheroids were detected in the EGFP/FITC channel using a set pixel size of 1.26 µm.

High-resolution spheroid imaging in glass bottom microwell plates

Spheroids were transferred from the microwell plate (Axonlab) to a flat glass-bottom plate (TPP) in FluoroBrite™ DMEM on day 4 post-spheroid formation and embedded into Cultrex extracellular matrix (Bio-Techne). Spheroid morphology and cancer cell invasion into the matrix was visualized after 24 hours by cell type-specific fluorescence with a Leica SP8 laser scanning microscope. 3D representations from Z-stacks were rendered in Imaris software (Oxford Instruments). Z-slices were stacked for 2D projections using Adobe Photoshop.

Metabolic flux analysis

Spheroids were transferred from a microwell plate (Axonlab) on Geltrex (Thermo Fisher) matrix-coated Seahorse cell culture plates on days 4, 8, and 10 after spheroid formation. BMDMs were harvested on day 7 from the temperature-responsive cell culture plates after cooling to room temperature and replated into Seahorse 24-well plates. The mitochondrial function (oxygen consumption rate) and glycolysis (acidification rate) of spheroids or BMDMs were measured using a Seahorse XF24 extracellular flux analyzer and the Cell Mito Stress Kit (Agilent Technologies) according to the instructions provided by the manufacturer.

3D cell viability assay

ATP concentration was measured in single spheroids with the CellTiter-Glo® 3D cell viability assay on days 4, 8, and 10 post-spheroid formation, according to the instructions provided by the manufacturer (Promega). Luminescence was measured with an infinite M200 Pro plate reader (Tecan).

Tissue/cell preparation and digestion

RBC isolation and RBC-ghost preparation

C57BL/6J mice were anesthetized with isoflurane, and blood was collected by cardiac puncture. RBCs were then washed, centrifuged (4000*g*, 30 min, 4°C), and resuspended in PBS. For RBC-ghost preparations, RBCs were lysed in 50 ml ultrapure water for 30 min on ice, centrifuged (3000*g*, 20 min, 4°C), and resuspended in PBS. The procedure was performed twice for each sample. The solutions were stored at 4°C for 12 hours.

Matrigel plug preparation and digestion

Matrigel (Corning) was thawed overnight on ice in a cold room (4°C) and mixed with RBCs (RBC-heme) or RBC-ghost membranes at a 1:1 ratio. In fibroblast growth factor (FGF)-enriched plugs, Matrigel was mixed with 500 ng/ml FGF (R&D Systems). Matrigel plugs were explanted seven days post-injection. Excised Matrigel plugs were mechanically disrupted, placed in pyruvate-free DMEM with 1.2 mM calcium chloride dihydrate and 1 mg/mL collagenase IV (Stemcell), and incubated on a shaker at 37°C for 60 min. Cells were passed through a 70 µm cell strainer, and RBCs were lysed with 1x RBC lysis buffer (BioLegend). The samples were then washed, centrifuged (450*g*, 5 min, 4°C), and resuspended in 1% FBS in PBS for flow cytometry measurement or in 0.1% bovine serum albumin (BSA) in 2 mM EDTA PBS for macrophage isolation and transcriptome analysis. For spectrophotometry with spectral-deconvolution and size exclusion chromatography (SEC), RBC-heme plugs were digested on days 1, 4, and 5 post-implantation, centrifuged (10000*g*, 5 min, 4°C), and the supernatant analyzed stored at -80 °C until analysis.

Macrophage isolation from digested Matrigel plugs

Anti-rat IgG Dynabeads (Invitrogen) were washed and incubated with rat anti-mouse F4/80 IgG2a antibodies (BD Biosciences) and CD11b IgG2b antibodies (BioLegend) at a ratio of 3.33 µg of antibody per 50 µl of Dynabeads. Single-cell suspensions from Matrigel plugs were incubated with anti-F4/80-coated (for RT-qPCR experiments) or anti-F4/80 and anti-CD11b-coated Dynabeads (for scRNA-seq experiment) on a shaker at 4°C for 30 min. After incubation, a positive selection of Dynabead-bound single-cell suspensions was performed on a DynaMag magnet (Invitrogen) with three washing steps, as suggested by the manufacturer's instructions.

Spheroid digestion

Spheroids were dissociated in 2 ml digestion medium (RPMI medium (Gibco) + 25 µg/ml Liberase ™ (Roche) + 40 µg/ml DNase I (Roche; 2000 U/ml) and incubated for 30-45 min in a water bath at 37°C with gentle shaking every 5 min. Then, 4 ml PBS + 0.04% BSA was added to stop the digestion. Digested spheroids were used immediately.

Spectrophotometry and spectral deconvolution

For spectral deconvolution of hemoglobin and hemoglobin metabolites (i.e., bilirubin), the absorption spectra of supernatants from digested RBC-heme Matrigel plugs and Matrigel alone were measured on a microvolume UV-Vis spectrophotometer (NanoDrop One, Thermo Fisher) using the manual mode. The absorption spectrum from 190 to 850 nm was acquired with a 0.5 nm resolution. We measured from each sample 2 µl according to the manufacturer's instructions. Then, the acquired absorption spectrum was deconvolved using reference spectra for oxy-hemoglobin, met-hemoglobin, and bilirubin based on a non-negative least-squares method (7). Before deconvolution, the biological background from the Matrigel was subtracted.

Size exclusion chromatography (SEC-HPLC)

After digestion, the supernatant was subjected to SEC to quantify free heme and hemoglobin in the RBC-heme plugs. Each supernatant was additionally measured after adding haptoglobin (CSL Behring) and hemopexin (CSL Behring) to assess the biologically available heme. Intact hemoglobin is complexed within haptoglobin, while biologically available heme is complexed within hemopexin, resulting in a mass shift and change in the elution profile. Therefore 200 µl of supernatant was mixed with 10 µl haptoglobin 1-1 (105 mg/ml), followed by 10 µl hemopexin (92 mg/ml). Then 10 µl of each sample was separated on a YMC-Pack Diol SEC column (4.6 mm ID, 30 nm, S-3 µm, 300 x 4.6 mm, DL30S03-3046WT) connected to a Gilson 307 HPLC Pump operated in an isocratic mode. Ammonium nitrate (0.2M, pH 7.4) with a flow of 0.5 ml/min was used as a mobile phase, and the elution profile was measured at 414 nm using a spectrophotometer (Jasco UV-970 Intelligent UV/VIS Detector) (8, 9).

Mouse models

Subcutaneous Matrigel plug model in mice

350 µl Matrigel mixture was injected subcutaneously into the flanks of anesthetized (intraperitoneal injection of ketamine (80 mg/kg), xylazine (16 mg/kg), acepromazine (3 mg/kg)) C57BL/6J mice using a 24-G needle. After seven days, mice were euthanized, and plugs were removed for downstream analysis.

For anti-CD40 antibody experiments, the mice were treated intravenously on day 7 with an agonistic anti-CD40 antibody (20 mg/kg, InVivoPlus, clone FGK4.5) or an isotype control antibody. Mice were euthanized after 24 hours, and plugs were collected. F4/80^{+} macrophages were recovered from digested Matrigel plugs, and gene expression was analyzed by RT-qPCR.

Lung metastasis model in mice

Approximately 750 spheroids were collected from microwell plates (equal to the content of one macro well) at different time points post-spheroid formation and injected intravenously into the tail vein of C57BL/6J or Rag2$^{-/-}$γc$^{-/-}$ mice. Three weeks postinjection, the lungs of anesthetized mice were perfused with PBS through the right ventricle and the trachea and collected for whole organ fluorescence imaging with a Zeiss Discovery V8 stereomicroscope and histology. For the metastasis experiments shown in Figure 9H, metastasis were manually counted to enhance sensitivity and specificity in the low disease burden range, to assess the effects of anti-CD40 treatment.

Tumor growth model in mice

Once confluent, GFP-MC38 tumor cells were harvested using 5 mM EDTA (Gibco) (4 min at 37°C) and washed twice in PBS. MC38 cells (2 x 10^{6}) in culture medium were mixed with Geltrex (Thermo Fisher) and injected subcutaneously into the mouse flanks. Agonistic anti-CD40 treatment (20 mg/kg, InVivoPlus, clone FGK4.5) or an isotype control antibody was administered intravenously seven days after tumor cell injection. In some experiments, animals were rechallenged two days after the first treatment. Mice were euthanized, and tumors were collected two or three days after antibody administration. GFP fluorescence was measured immediately, and tumors were then fixed in formalin (10%) and stored at room temperature.

Flow cytometry

Cells were preincubated with Mouse BD Fc Block™ (≤ 1 µg/million cells in 100 µl, BD Biosciences) at 4°C for 10 min. The following antibodies were purchased from BD Biosciences: anti-CD45 (clone 30-F11), anti-F4/80 (clone T45-2342), and anti-I-A/I-E (clone M5/114.15.2). Anti-CD11b (clone M1/70) was purchased from BioLegend. Single-color controls for spectral unmixing were prepared with Ultracomp beads (Thermo Fisher). Multiparameter analysis was performed with an Aurora 5L spectral flow cytometer (Cytek). The data were analyzed using FlowJo software (version 10.7.1).

Histology

Organ fixation for paraffin embedding and microtome sectioning

Mice were anesthetized by intraperitoneal injection of ketamine (80 mg/kg), xylazine (16 mg/kg), and acepromazine (3 mg/kg) and transcardially perfused with cold PBS. Organs were placed in 10% formalin and transferred in 70% ethanol after 24 hours before embedding in paraffin blocks. Microtome sections (2-2.5 µm) of each organ were cut for H&E staining, Perl's staining, immunohistochemistry, or immunofluorescence staining.

Immunohistochemistry and iron staining

Nonheme iron staining: Tissue paraffin sections were incubated with Perl's iron reagent containing 5% potassium ferrocyanide and 2% hydrochloric acid for 60 min at room temperature, after which they were rinsed in deionized water. Sections were then incubated with 3% hydrogen peroxide and methanol for 20 min at room temperature.

GFP staining: Tissue sections were incubated overnight with a goat anti-GFP antibody (Abcam) diluted 1:1000, followed by a biotinylated horse anti-goat secondary antibody (Vector) diluted 1:500. All immunohistochemical sections were rinsed in 0.1 M phosphate buffer, pH 7.4, and incubated with diaminobenzidine (DAB, Abcam) for 2-5 min. After incubation, sections were washed in deionized water and lightly counterstained with Gill No. 2 hematoxylin (Sigma).

Multiplexed immunofluorescence staining: Paraffin-embedded microtome sections were stained for immunofluorescence analysis using the Opal 4-Color anti-Rabbit Manual IHC Kit (Akoya Biosciences) as instructed by the manufacturer. The following primary antibodies were used: rabbit anti-mouse F4/80 antibody solution (1:1000, Cell Signaling), rabbit anti-mouse HMOX-1

(1:500, Enzo LifeScience), and rat anti-mouse TER-119 (1:500, eBioscience). After the first antibody incubation, the slides were washed in TBST (1x TBS + 0.05% Tween 20 in PBS) and incubated for 10 min with secondary Opal Polymer anti-Rabbit HRP (Akoya Biosciences) diluted 1:5 in Opal Polymer anti-Rabbit HRP Diluent (Akoya Biosciences) or anti-rat HRP (Thermo Fisher) diluted 1:200 in Opal Polymer anti-Rabbit HRP Diluent (Akoya Biosciences). The slides were washed again in TBST and incubated for 10 min with Opal Fluorophore Working solution (1:100 dilution of fluorophore in Amplification Diluent, Akoya Biosciences). After washing with TBST, the slides were placed in boiling pH 6 AR buffer (Akoya Biosciences) for 30 min to remove the bound antibodies and then allowed to cool for 15 min. The slides were counterstained with spectral DAPI solution (Akoya Biosciences) and mounted using ProLong Gold Antifade Mountant (Thermo Fisher).

Microscopy image acquisition and analysis

Whole-lung sections and subcutaneous tumors were imaged with a Zeiss Axio Scan Z1 Slidescanner microscope. Multiplexed immunofluorescence images were acquired with an Akoya Vectra Polaris/PhenoImager HT. Whole spheroids were visualized with a Leica SP8 confocal laser scanning microscope. Images were analyzed using Qupath and ImageJ. Brightness, contrast, and color tone (for single-channel fluorescence images) were adjusted with Adobe Lightroom software version 6.3.1. using identical settings for all images of an experiment.

Visium CytAssist Spatial Gene Expression for FFPE

Subcutaneous mouse tumors were processed as described in the section titled organ fixation for paraffin embedding and microtome sectioning. Microtome sections on regular glass slides were deparaffinized and stained for H&E as described in Visium CytAssist Spatial Gene Expression for FFPE (10X Genomics, CG000520). Regions of interest on the tumor sections were chosen using H&E staining and aligned in the tissue slide cassette 6.5 mm. After destaining and decrosslinking, sections were immediately subjected to probe hybridization overnight followed by probe ligation, release and extension and spatial library construction as described in Visium CytAssist Spatial Gene Expression for FFPE (10X Genomics, CG000495). Ready-made libraries were sequenced at the Functional Genomics Center Zurich (FGCZ) on an Illumina NovaSeq 6000 system.

Sequencing-based workflows and data analysis

Bulk RNA sequencing

RNA was extracted from BMDMs using the RNeasy Micro kit (Qiagen) according to the manufacturer's protocol, including on-column DNase I treatment. RNA quality was validated with an Agilent Technologies 2100 Bioanalyzer using an RNA chip, and only samples with an RNA integrity number (RIN) of > 9 were used for sequencing. cDNA libraries were generated from the RNA samples using the Illumina TruSeq RNA stranded kit following the manufacturer's instructions. Libraries were amplified by PCR (total of 15 cycles), and the quality and concentrations of the libraries were determined using an Agilent Fragment Analyzer with DNA High-Sensitivity Chips. The libraries were pooled in equimolar amounts and sequenced in an Illumina NovaSeq 6000 sequencer (single-end 100 bp) with a depth of approximately 20 million reads per sample.

Bulk RNA sequencing data analysis

Reads were aligned to the reference genome Ensembl GRCm38.p5 Release 91 using STAR (v2.7.0e) (10). The quality of alignment was evaluated using Samtools (v1.9) (11). Counts were obtained using the featureCounts function of the Rsubread package (v1.22.2)(12). Differential expression analysis was performed with the DESeq2 R package (v1.26.0)(13).

scRNA-seq sample preparation

Single-cell suspensions were multiplexed according to the experimental design and then used for GEM generation following the 10X Genomics protocol (CG000388 Demonstrated Protocol Chromium Next GEM Single Cell 3' v3.1 (Dual Index) with Feature Barcode technology for Cell Multiplexing Rev B) targeting a cell recovery rate of 10,000 cells. GEM generation and library preparation were performed according to the 10X Genomics protocol (CG000388). Ready-made libraries were sequenced at the Functional Genomics Center Zurich (FGCZ) on an Illumina NovaSeq 6000 system. Downstream analysis was performed in Python (version 3.8.6) with Scanpy (1.7.2) (14).

scRNA-seq sample analysis

Read alignment

Reads were aligned to the mouse reference genome Ensembl GRCm39 (Release_106-2022-07-05) using CellRanger (version 7.0.0).

Quality Control and Preprocessing

To assess the quality of the cells, the following covariates were considered: number of genes expressed in a cell (*n_genes_by_counts*), number of counts per cell (*total_counts*), and percentage of mitochondrial RNA (*pct_counts_mt*). Cells that expressed fewer than *min_genes* or more than *max_genes* were filtered out. Cells with a percentage of mitochondrial RNA greater than *max_pct_mt* were considered dead and removed from the analysis. Genes that were expressed by fewer than *min_cells* cells were excluded. See below for the cutoff values used in each experiment. The count data were normalized by an algorithm based on deconvolving size factors from cell pools implemented in the R package scran (`calculateSumFactors`)(15) and log(x+1) (`sc.pp.log1p`) transformed, yielding normalized expression values.

Data integration

Multiplexed samples were merged into one dataset by simple concatenation. Additionally, samples from different experiments were integrated using the harmony algorithm (`sc.external.pp.harmony_integrate`) (16) after normalization and PCA.

Dimensionality reduction and clustering

For dimension reduction, the following steps were performed using the Python package Scanpy: identifying highly variable genes (`sc.pp.highly_variable_genes`), performing PCA using highly variable genes (`sc.tl.pca`), computing the neighborhood graph (`sc.pp.neighbors`) and computing the UMAP (`sc.tl.umap`). The cells were clustered using Leiden clustering (`sc.tl.leiden`), which depends on the neighborhood graph. The resolution of the Leiden clustering was chosen so that a biologically meaningful number of clusters was produced.

Cell type annotation and functional classification

To identify cell types, we analyzed the expression of marker genes and other differentially expressed genes (`sc.tl.rank_genes_groups` with `method = 'wilcoxon'`).

Depending on the experiment, we analyzed these genes separately or by scoring gene sets (`sc.tl.score_genes`). GSEA was performed to assess functional and biological process-related differences between clusters or conditions. First, genes were ranked using the output of the Wilcoxon rank-sum test (`rank = -log10(adj. p value)*sign(logfoldchange)`) and then fed to the GSEA algorithm implemented in the Python package gseapy (17) (`gseapy.prerank`), resulting in a normalized enrichment score (NES) and a false discovery rate (FDR) per gene set. For transcription factor analysis, `gseapy.enrichr` with the TRRUST_Transcription_Factors_2019 gene set database was used.

Spatial transcriptomics analysis

Downstream analysis was performed in Python (version 3.9.13) with Scanpy (1.9.1) (14). Each sample was processed individually.

<u>Read alignment</u>

Reads were aligned to the mouse reference genome Ensembl GRCm39 (Release_106-2022-07-05) using SpaceRanger (version 2.1.0).

<u>Quality Control and Preprocessing</u>

Genes that were detected in fewer than 50 spots were excluded from further analysis. Counts were normalized (`sc.pp.normalize_total`) and log(x+1) (`sc.pp.log1p`) transformed.

<u>Tumor segmentation</u>

Clustering (`sc.pp.neighbors, sc.tl.leiden`) and differential gene expression of the clusters (`sc.tl.rank_genes_groups` with `method = 'wilcoxon'`) was performed to identify tumor cells. The projection of the identified tumor clusters on the spatial image yielded the tumor outline.

<u>Oxidative stress and Nrf2 scoring</u>

Spots were scored (`sc.tl.score_genes)` for oxidative stress using a gene set extracted from the list of differentially expressed genes of the RBC-heme plug (cutoffs: log2FC > 2, adj. p-val < 0.001). Genes that are activated by NRF2 were obtained from the TRRUST version 2 database (18).

Figure-by-figure details of sample preparation and data analysis (sequencing data)

Sample preparation for scRNA-seq

Experiment related to Figure 3

Macrophage-enriched single-cell suspensions from RBC-heme and RBC-ghost Matrigel plugs were used for GEM generation following 10X Genomics protocol (CG000388 Demonstrated Protocol Chromium Next GEM Single Cell 3' v3.1 (Dual Index) with Feature Barcode technology for Cell Multiplexing Rev B) targeting a cell recovery rate of 10’000 cells for each condition.

Experiment related to Figure 4

Mixed cell-type spheroids of GFP-MC38 cancer cells and BMDMs that were pretreated with either IFNy, LPS, heme + IFNy , heme + LPS, or heme were grown in microwell plates for 24 hours. 9000 spheroids per condition were pooled and digested for 10 min in a water bath at 37°C with gentle shaking and subsequently resuspended using a 1 ml pipet to disrupt the digested spheroids into single cells. Cells were centrifuged at 300*g* for 5 min at 4°C and resuspended with PBS + 0.04% BSA. Single-cell suspensions were counted and transferred to 2 ml safe lock tubes for cell staining using lipid tags following 10X Genomics protocol (CG00391; Demonstrated Protocol Cell Multiplexing Oligo Labeling for Samples with >80% Viable Cells; Rev B). Cell Multiplexing Oligos (CMO) labels B301 - B305 (3’ CellPlex Kit Set A) were used to label the samples. After labeling, cell suspensions were counted again and the samples were pooled according to the pooling calculations in appendix of the labeling protocol (CG00391) in equivalent ratios. The pool was then directly used for GEM generation following 10X Genomics protocol (CG000388 Demonstrated Protocol Chromium Next GEM Single Cell 3' v3.1 (Dual Index) with Feature Barcode technology for Cell Multiplexing Rev B) targeting a cell recovery rate of 10’000 cells.

Experiment related to Figure 5

Spheroids of GFP-MC38 and GFP-MC38 mixed with BMDMs that were pretreated with heme were grown in microwell plates for four, eight, and ten days. 9000 spheroids per condition were pooled and digested as aforementioned for 40 min in a water bath at 37°C with gentle shaking and subsequently resuspended using a 1 ml pipet to disrupt the digested spheroids into single cells. Cells were centrifuged at 300*g* for 5 min at 4°C and resuspended with PBS + 0.04% BSA. Single-cell suspensions were counted and transferred to 2 ml safe lock tubes and directly used

for GEM generation following 10X Genomics protocol (CG000388 Demonstrated Protocol Chromium Next GEM Single Cell 3' v3.1 (Dual Index) with Feature Barcode technology for Cell Multiplexing Rev B) targeting a cell recovery rate of 10’000 cells.

Experiment related to Figure 6

Spheroids of GFP-MC38 and GFP-MC38 mixed with unstimulated BMDMs or BMDMs that were pretreated with either IFNγ, heme + IFNγ, or heme were grown in microwell plates for nine days. 4500 spheroids per condition were pooled and digested as aforementioned for 45 min in a water bath at 37°C with gentle shaking and subsequently resuspended using a 1 ml pipet to disrupt the digested spheroids into single cells. Cells were centrifuged at 300*g* for 5 min at 4°C and resuspended with PBS + 0.04% BSA. Single-cell suspensions were counted and transferred to 2 ml safe lock tubes for cell staining using lipid tags following 10X Genomics protocol (CG00391; Demonstrated Protocol Cell Multiplexing Oligo Labeling for Samples with >80% Viable Cells; Rev B). Cell Multiplexing Oligos (CMO) labels B301 - B305 (3’ CellPlex Kit Set A) were used to label the five samples. After labeling, cell suspensions were counted again, and the five samples were pooled according to the pooling calculations in appendix of the labeling protocol (CG00391) in equivalent ratios. The pool was then directly used for GEM generation following 10X Genomics protocol (CG000388 Demonstrated Protocol Chromium Next GEM Single Cell 3' v3.1 (Dual Index) with Feature Barcode technology for Cell Multiplexing Rev B) targeting a cell recovery rate of 10’000 cells.

Experiment related to Figure 7

BMDMs from one conditional *Keap1* KO mouse (control) and one WT littermate mouse (control and heme-treated) were resuspended and transferred into 15 ml falcon tubes. Tubes were filled up with RPMI 1% BSA + 2mM EDTA and centrifuged at 300*g* for 10 min at room temperature. The supernatant was discarded, and cells were taken up in 1 ml RPMI + 1% BSA + 2mM EDTA for counting. 1 x 10^6 cells per condition were taken for cell staining using lipid tags following 10X Genomics protocol (CG00391; Demonstrated Protocol Cell Multiplexing Oligo Labeling for Samples with >80% Viable Cells; Rev B). Cell Multiplexing Oligos (CMO) labels B308 - B311 (3’ CellPlex Kit Set A) were used to label the four samples.

After labeling, cell suspensions were counted again, and the samples were pooled according to the pooling calculations in the appendix of the labeling protocol (CG00391) in equivalent ratios.

The pool was then directly used for GEM generation following 10X Genomics protocol (CG000388 Demonstrated Protocol Chromium Next GEM Single Cell 3' v3.1 (Dual Index) with Feature Barcode technology for Cell Multiplexing Rev B) targeting a cell recovery rate of 10’000 cells.

GEM generation and library preparation were performed for all experiments according to 10X Genomics protocol (CG000388). Ready-made libraries were sequenced at the Functional Genomics Center Zurich (FGCZ) on an Illumina NovaSeq 6000 system following the recommendations of 10X Genomics.

scRNA-seq data analysis

<u>Experiment related to Figure 3</u>

Preprocessing, Dimensionality reduction, Clustering

Functional annotation of clusters with GSEA (both conditions) (Fig. 3B-C), visualization with scoring of functional gene sets (per condition) (Supplementary Fig. 1C)

Visualization of expression values of marker genes (Fig. 3D)

<u>Experiment related to Figure 4</u>

Preprocessing, Dimensionality reduction, Clustering

Extract clusters of macrophages by means of marker gene expression (Fig. 4G)

Recompute highly variable genes and PCA in macrophages (Fig. 4H), visualize PC1 loadings (Fig. 4I)

Visualization of expression values of marker genes (Fig. 4J)

<u>Experiment related to Figure 5</u>

Separate processing of these two conditions

Preprocessing, Dimensionality reduction, Clustering

Functional classification of clusters by means of DGE and GSEA using the MSigDB_Hallmark_2020 database (Fig. 5E)

Visualization of distribution of cell types by age of spheroids with normalized density embedding (`sc.tl.embedding_density, sc.pl.embedding_density`) (Fig. 5D)

Visualization of expression values of marker genes (Supplementary Fig. 3E)

Experiment related to Figure 6

Preprocessing, Dimensionality reduction, Clustering

Functional classification of clusters by means of DGE and GSEA using the MSigDB_Hallmark_2020 database (Fig. 6E)

Visualization of the functional class by scoring gene sets (Fig. 6G)

Experiment related to Figure 7

Preprocessing, Dimensionality reduction

Visualization of expression values of marker genes (Fig. 7E)

Transcription Factor Analysis of PCA loadings using the (Fig. 7F)

Table 1: Sequences for PCR primers

Target gene	Forward sequence (5′-3′)	Reverse sequence (3′-5′)	
Arg1	GTAGACCCTGGGGAACACTAT	ATCACCTTGCCAATCCCCAG	
Cd74	CACCGAGGCTCCACCTAAAG	TTACCGTTCTCGTCGCACTT	
Gclm	AGTTGACATGGCATGCTCCG	CCATCTTCAATCGGAGGCGA	
Gstm1	GAACCAGGTCATGGACACCC	GCAATGGAACAGCCACAAAGT	
H2-Ab1	ACGGTGTGCAGACACAACTA	CGACATTGGGCTGTTCAAGC	
H2-Eb1	ACGGTGTGCAGACACAACTA	GTCACCGTAGGCTCAACTCT	
Hmox1	AGGCTAAGACCGCCTTCCT	TGTGTTCCTCTGTCAGCATCA	
Marco	TTCTGTCGCATGCTCGGTTA	CAGATGTTCCCAGAGCCACC	
Nqo1	AGCGTTCGGTATTACGATCC	AGTACAATCAGGGCTCTTCTCG	
Prdx1	TGTCCCACGGAGATCATTGC	GGGTGTGTTAATCCATGCCAG	
Slc40a1	GGCACTTTGCAGTGTCTGTG	GTGACGTCTGGGCCACTTTA	
Slc48a1	CTTCTTCGTGGGTGCTCTCT	GTTCGGGTCTTTGAGACTCTG	
Slc7a11	GATTCATGTCCACAAGCACAC	GAGCATCACCATCGTCAGAG	
Spp1	CCTGGCTGAATTCTGAGGGAC	ATCAGTCACTTTCACCGGGAG	
Cxcl9	CTTTTCCTCTTGGGCATCAT	GCATCGTGCATTCCTTATCA	
Cxcl10	GCTGCCGTCATTTTCTGC	TCTCACTGGCCCGTCATC	

Table 2: Parameters for scRNA-seq analysis

Figure	Experiment	min_genes	max_genes	max_pct_mt	min_cells	max_pct_Rp	min_counts	max_counts	data integration with harmony
Fig. 3		500	8000	12.5	20	NA	5000	80000	no
Fig.4		500	10000	15	20	NA	NA		no
Fig. 5		2000	10000	15	20	45	NA		no
Fig. 6		500	10000	12	20	NA	NA		no
Fig. 7		1500	8000	15	20	NA	NA	NA	no

REFERENCES

1. Goldman MJ, et al. Visualizing and interpreting cancer genomics data via the Xena platform. *Nat Biotechnol*. 2020;38(6):675–678.

2. Taguchi K, et al. Genetic analysis of cytoprotective functions supported by graded expression of Keap1. *Mol Cell Biol*. 2010;30(12):3016–3026.

3. Kong X, et al. Enhancing Nrf2 pathway by disruption of Keap1 in myeloid leukocytes protects against sepsis. *Am J Respir Crit Care Med*. 2011;184(8):928–938.

4. Reddy NM, et al. Conditional deletion of Nrf2 in airway epithelium exacerbates acute lung injury and impairs the resolution of inflammation. *Am J Respir Cell Mol Biol*. 2011;45(6):1161–1168.

5. Vallelian F, et al. Revisiting the putative role of heme as a trigger of inflammation. *Pharmacol Res Perspect*. 2018;6(2):e00392.

6. Bankhead P, et al. QuPath: Open source software for digital pathology image analysis. *Sci Rep*. 2017;7(1):16878.

7. Deuel JW, et al. Different target specificities of haptoglobin and hemopexin define a sequential protection system against vascular hemoglobin toxicity. *Free Radic Biol Med*. 2015;89:931–943.

8. Buzzi RM, et al. Modular Platform for the Development of Recombinant Hemoglobin Scavenger Biotherapeutics. *Mol Pharm*. 2021;18(8):3158–3170.

9. Buehler PW, et al. Structural stabilization in tetrameric or polymeric hemoglobin determines its interaction with endogenous antioxidant scavenger pathways. *Antioxid Redox Signal*. 2008;10(8):1449–1462.

10. Dobin A, et al. STAR: ultrafast universal RNA-seq aligner. *Bioinformatics*. 2013;29(1):15–21.

11. Danecek P, et al. Twelve years of SAMtools and BCFtools. *Gigascience*. 2021;10(2):giab008.

12. Liao Y, Smyth GK, Shi W. The R package Rsubread is easier, faster, cheaper and better for alignment and quantification of RNA sequencing reads. *Nucleic Acids Res*. 2019;47(8):e47.

13. Love MI, Huber W, Anders S. Moderated estimation of fold change and dispersion for RNA-seq data with DESeq2. *Genome Biol*. 2014;15(12):550.

14. Wolf FA, Angerer P, Theis FJ. SCANPY: large-scale single-cell gene expression data analysis. *Genome Biol*. 2018;19(1):15.

15. Lun ATL, McCarthy DJ, Marioni JC. A step-by-step workflow for low-level analysis of single-cell RNA-seq data with Bioconductor. *F1000Res*. 2016;5:2122.

16. Korsunsky I, et al. Fast, sensitive and accurate integration of single-cell data with Harmony. *Nat Methods*. 2019;16(12):1289–1296.

17. Fang Z, Liu X, Peltz G. GSEApy: a comprehensive package for performing gene set enrichment analysis in Python. *Bioinformatics*. 2023;39(1):btac757.

18. TRRUST - download [Internet]https://www.grnpedia.org/trrust/downloadnetwork.php. Accessed June 26, 2023
